# Genetically Engineered Probiotic Nanoplatform for Ultrasound‐Enhanced Colorectal Cancer Therapy via Targeted Delivery, Immune Modulation, and Anti‐Angiogenesis

**DOI:** 10.1002/mco2.70871

**Published:** 2026-07-16

**Authors:** Jie Long, Tongrui Shang, Xuezhong Mo, Yunfang Yu, Feng Lin, Zixuan Liang, Olivia Monteiro, Daniel Baptista‐Hon, Dominic Chi‐Chung FOO, Lui Ng, Renchuan Liang, Man Tong, Weizhong Tang, Kang Zhang, Yunxi Huang

**Affiliations:** ^1^ Department of Otolaryngology‐Head and Neck Surgery, Colorectal and Anal Disease Unit, Guangxi Key Laboratory of Basic and Translational Research for Colorectal Cancer Guangxi Medical University Cancer Hospital Nanning China; ^2^ Guangdong Provincial Key Laboratory of Malignant Tumor Epigenetics and Gene Regulation Guangdong‐Hong Kong Joint Laboratory for RNA Medicine Sun Yat‐sen Memorial Hospital, Sun Yat‐sen University Guangzhou China; ^3^ Artificial Intelligence Cross Disciplinary Research Institute, Faculty of Medicine, Faculty of Innovation Engineering, School of Computer Science and Engineering Macau University of Science and Technology Macau China; ^4^ Guangdong Provincial Key Laboratory of Cancer Pathogenesis and Precision Diagnosis and Treatment, AI Big Data Laboratory, Shenshan Medical Center Memorial Hospital of Sun Yat‐Sen University Shanwei China; ^5^ The First Affiliated Hospital, Jinan University Guangzhou China; ^6^ School of Medicine University of Dundee Dundee UK; ^7^ Department of Surgery University of Hong Kong Hong Kong, SAR China; ^8^ School of Biomedical Sciences The Chinese University of Hong Kong Hong Kong SAR China; ^9^ State Key Laboratory of Eye Health, National Clinical Research Center for Ocular Diseases Eye Hospital and Institute for Advanced Study On Eye Health and Diseases, Wenzhou Medical University Wenzhou China; ^10^ The first Afflaited Hospital Chongqing Medical University Chongqing China; ^11^ Guangzhou National Laboratory Guangzhou China

**Keywords:** acoustic reporter genes, chinese medicine curcumin, colorectal cancer, *Escherichia coli* Nissle 1917, tumor microenvironment

## Abstract

Colorectal cancer (CRC) remains a leading cause of cancer‐related mortality worldwide, largely due to limitations in early diagnosis and effective treatment. To address these challenges, we developed a multifunctional probiotic nanoplatform, EcNA@Cur‐Lip‐FA, that integrates real‐time diagnosis, targeted therapy, and immune modulation for enhanced CRC treatment. Specifically, this system leverages genetically engineered *Escherichia coli* Nissle 1917 (EcN) encoding acoustic reporter genes (ARGs) to produce ultrasound (US)‐responsive gas vesicles (GVs), enabling real‐time, noninvasive tumor imaging. Simultaneously, folic acid (FA)‐modified curcumin‐loaded liposomes (Cur‐Lip‐FA) are co‐delivered with EcN, enhancing tumor‐specific accumulation and therapeutic precision. After being taken up by tumor cells, curcumin is released locally upon exposure to US irradiation, generating reactive oxygen species (ROS), inducing immunogenic cell death (ICD), and activating the cGAS‐STING pathway to promote dendritic cells (DCs) maturation and robust CD4^+^/CD8^+^ T‐cell responses. Additionally, EcNA@Cur‐Lip‐FA downregulates angiogenesis‐related factors (VEGF, VEGFR1, and ANGPT1), thereby inhibiting tumor neovascularization. In vitro and in vivo studies demonstrated that EcNA@Cur‐Lip‐FA enables precise tumor localization, dynamic imaging, and effective suppression of both primary CRC and metastatic liver lesions with favorable biocompatibility. This multifunctional platform offers a promising strategy for integrating diagnosis and treatment, paving the way for next‐generation CRC theranostics.

## Graphical abstract

The study constructed an integrated diagnosis‐treatment system by using engineered probiotic EcN expressing ARGs and Cur‐Lip‐FA. It enables US guided tumor targeting, real‐time in vivo drug monitoring, and US‐assisted curcumin induced immunogenic cell death, and activating the cGAS‐STING pathway to boost CD4^+^/CD8^+^ T cell anti‐tumor immunity.

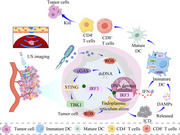

## 1 Introduction

Colorectal cancer (CRC) remains one of the most prevalent and deadly cancers globally [1, 2]. Therefore, early diagnosis and treatment are paramount for improving prognosis and survival rates. Current diagnostic tools for CRC primarily depend on colonoscopy, yet this method is inconvenient, costly, and linked to rare but serious complications [3]. Ultrasound (US) imaging, recognized for its non‐invasive, real‐time, and repeatable nature, offers a promising approach for dynamically monitoring CRC growth and metastasis [4]. However, the application of US in CRC diagnosis is often hindered by interference from intestinal gas, which limits its ability to assess tumor morphology and invasion depth accurately [5]. Therefore, the development of novel real‐time diagnostic strategies for CRC, coupled with the exploration of synergistic multimodal treatment approaches, holds significant promise for improving the clinical outcomes of patients.

Recently, synthetic biology tools, which can be leveraged to enable orally delivered bacteria to encode and locally deliver diagnostic and therapeutic molecules to intestinal lesions, have garnered increasing attention [6]. Notably, acoustic reporter genes (ARGs) can be integrated into the body of microbes through genetic engineering techniques to emit detectable signals under US stimulation, which not only achieves noninvasive imaging of tumors but also significantly enhances the accuracy and sensitivity of diagnosis [7, 8, 9]. Typically, *Escherichia coli* Nissle 1917 (EcN), a probiotic strain with favorable biosafety properties and beneficial effects on gut health, has been increasingly recognized as a promising platform for the expression of ARGs [10, 11]. By incorporating these ARGs, EcN produces US‐responsive signals, facilitating real‐time tumor monitoring and enhancing diagnostic precision [12].

Beyond early diagnosis, timely treatment strategies are crucial for eradicating small cancer lesions, inhibiting early tumor metastasis, and improving the overall prognosis [13]. Among them, antitumor strategies based on reactive oxygen species (ROS) have demonstrated significant potential for CRC treatment. Current ROS‐inducing CRC treatments [14], such as photodynamic therapy, chemotherapy, and redox modulation, face several challenges, including systemic toxicity, treatment site limitations, and suboptimal therapeutic efficacy [15, 16]. Given the pivotal role of intracellular ROS in antitumor effects, novel approaches targeting ROS have garnered significant interest. One such promising strategy involves curcumin, a naturally occurring polyphenol with strong ROS‐generating capacity and an excellent safety profile [17]. Additionally, curcumin can induce ICD and activate the cyclic GMP‐AMP Synthase—Stimulator of Interferon Genes (cGAS‐STING) pathway [18], orchestrating innate and adaptive immunity. Interestingly, curcumin also functions as a sonosensitizer, potentiating its therapeutic effects under US exposure [19]. Despite these promising characteristics, the widespread clinical application of curcumin is limited by its poor solubility, stability, and bioavailability [20, 21]. To address these challenges, curcumin liposomes (Cur‐Lip) have been developed, encapsulating curcumin in a lipid bilayer structure [22]. Additionally, conjugating curcumin liposomes with folic acid (FA) can further optimize drug accumulation in tumor tissues, with a lower incidence of toxic side effects, thereby amplifying its therapeutic effects [22].

Inspired by this, our study introduces an integrated diagnosis and treatment system, EcNA@Cur‐Lip‐FA, which combines genetically engineered EcNA (expressing ARGs) with Cur‐Lip conjugated to FA. In this system, the FA conjugates guide the EcNA@Cur‐Lip‐FA particles to accumulate specifically at the tumor site, enabling efficient long‐term drug delivery. Concurrently, the ARGs within EcN produce gas vesicles (GVs) that serve as US contrast agents, allowing for real‐time tumor monitoring. Under the acidic conditions of the TME, curcumin is released from the EcNA@Cur‐Lip‐FA system, inducing ICD and activating the cGAS‐STING pathway. This process promotes the maturation of DCs and enhances the activation of tumor‐killing CD4^+^ and CD8^+^ T cells. Both in vitro and in vivo experiments demonstrated that EcNA@Cur‐Lip‐FA can precisely target tumor sites, enable dynamic tumor monitoring, and effectively suppress both primary tumors and liver metastases. These findings suggest that our integrated diagnosis and therapy platform holds significant potential for revolutionizing CRC treatment by combining diagnostic and therapeutic modalities in a single, efficient system.

## 2 Results

### 2.1 Preparation and Characterization of EcNA@Cur‐Lip‐FA

**FIGURE 1:**
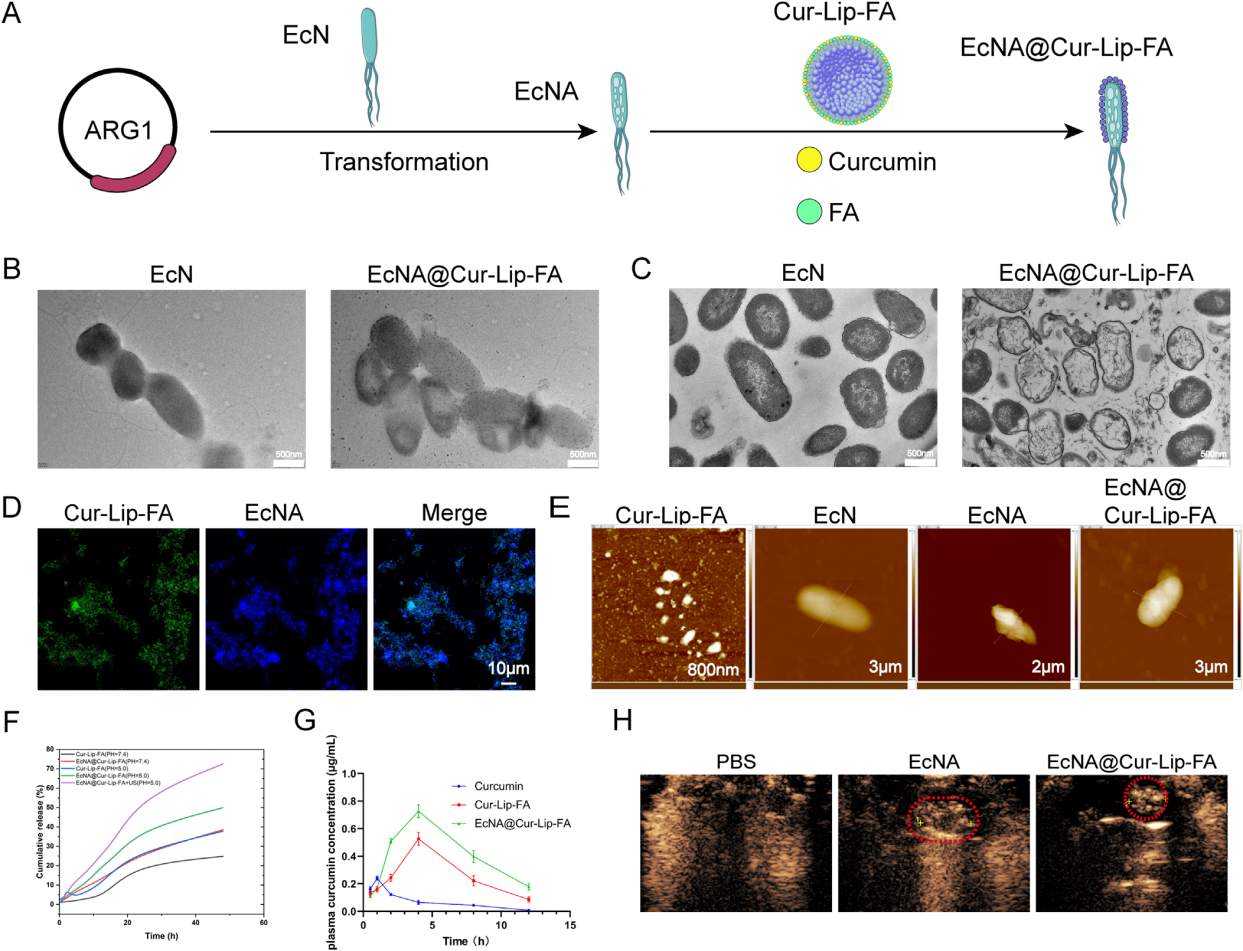
Synthesis and characterization of EcNA@Cur‐Lip‐FA. (A) Synthesis scheme of EcNA@Cur‐Lip‐FA. (B) TEM images of EcN and EcNA@Cur‐Lip‐FA (scale bar: 500 nm) (*n* = 3). (C) Bacterial section images of EcN and EcNA@Cur‐Lip‐FA (scale bar: 500 nm) (*n* = 3). D) Co‐localization imaging confirming surface conjugation of Cur‐Lip‐FA (DiO: green) with EcNA (DAPI: blue) (*n* = 3). (E) Atomic force microscope images of different NPs (*n* = 3). (F) Cumulative release curve of curcumin of Cur‐Lip‐FA and EcNA@Cur‐Lip‐FA at pH 5.0 and pH 7.4, respectively (*n* = 3). G) Plasma concentration–time curve of curcumin in plasma after oral administration (*n* = 3). (H) Ultrasound imaging of control, EcNA, and EcNA@Cur‐Lip‐FA (*n* = 3). The statistical method adopted ANOVA analysis. Data are presented as the mean ± SD. *p < 0.05, **p < 0.01, and ***p < 0.001.

EcNA@Cur‐Lip‐FA preparation is shown in Figure 1A. First, the bARG1 plasmid was transformed into EcN to generate EcNA—bacteria expressing GVs. As shown in Figure S1, gel electrophoresis demonstrated the successful expression of the ARGs in EcN (EcNA), as evidenced by the appearance of the target band in the third lane. To further support the successful construction of EcNA, centrifugation experiments revealed that EcN settled completely at the bottom of the tube, while EcNA floated on the surface of the liquid due to the presence of GVs (Figure S2).

To prepare EcNA@Cur‐Lip‐FA, we first employed the classical thin‐film method to create curcumin‐loaded liposomes conjugated with FA (Cur‐Lip‐FA). Western blot (WB) confirmed FOLR1 overexpression in CT26 tumor cells versus NCM460 normal intestinal cells (Figure S3), supporting folate‐receptor mediated targeting. Subsequently, EcNA and Cur‐Lip‐FA were incubated at room temperature for 2 h to form EcNA@Cur‐Lip‐FA nanoparticles (NPs). The transmission electron microscopy (TEM) images (Figure 1B) revealed that, compared to the EcN group, the morphology of EcNA@Cur‐Lip‐FA remained similar, with white GVs visible inside the bacteria. TEM of thin sections (Figure 1C) revealed vesicular structures in EcNA@Cur‐Lip‐FA, confirming the formation of NPs. UV‐Vis spectroscopy (Figure S4) showed a characteristic 425 nm absorption peak for Cur‐Lip‐FA, EcN@Cur‐Lip‐FA, and EcNA@Cur‐Lip‐FA, verifying Cur‐Lip‐FA incorporation. Yielded a curcumin loading of 5.8% (w/w), exceeding previously reported methods [23]. Colocalization analysis was conducted by labeling Cur‐Lip‐FA with DiO (green) and EcNA with DAPI (blue). As shown in Figure 1D, the overlap of green and blue signals further verified the successful preparation of EcNA@Cur‐Lip‐FA. To evaluate the morphology of the bacteria, atomic force microscopy (AFM) was employed. As shown in Figure 1E, EcN exhibited a typical rod‐like shape with a smooth surface. After the transformation and expression of ARGs, EcNA retained its rod shape but exhibited increased surface roughness and deformation. In contrast, the surface of EcNA@Cur‐Lip‐FA became notably rougher, with spherical structures visible, likely due to the adsorption of liposomes onto the bacterial surface and the expression of proteins. The dynamic light scattering (DLS) analysis (Figure S5) revealed that EcNA had a particle size of 2299.8 nm, while Cur‐Lip‐FA exhibited a smaller average particle size of 147.9 nm. After modification with Cur‐Lip‐FA, the particle size of EcNA@Cur‐Lip‐FA significantly increased to 3085.7 nm, with a zeta potential of −25.2 mV (Figure S6).

As depicted in Figure 1F, the cumulative release of curcumin from EcNA@Cur‐Lip‐FA + US at pH 5.0 over 48 h was approximately 80%, significantly higher than the release rate without US and at pH 7.4. This demonstrated that EcNA@Cur‐Lip‐FA can achieve pH‐ and US‐responsive controlled release. Next, we evaluated the pharmacokinetics of curcumin following the oral administration of EcNA@Cur‐Lip‐FA and Cur‐Lip‐FA in mice. The plasma concentration–time curves of curcumin are shown in Figure 1G. Compared to the curcumin suspension alone, both Cur‐Lip‐FA and EcNA@Cur‐Lip‐FA resulted in a significant increase in the plasma concentration of curcumin following oral administration. Notably, EcNA@Cur‐Lip‐FA exhibited the optimal pharmacokinetic profile, which can be attributed to EcN's ability to colonize the intestine, thereby enabling the sustained release of curcumin over time. Finally, we assessed the diagnostic capability of EcNA@Cur‐Lip‐FA for CRC. As illustrated in Figure 1H, US imaging revealed that EcNA@Cur‐Lip‐FA showed more distinct tumor boundaries compared to the control, suggesting enhanced tumor detection. Furthermore, Figure S7A also verified that EcNA@Cur‐Lip‐FA exhibited good targeting in tumor‐bearing mice. Quantitative data showed the same results (Figure S7B). Figure S8 further confirmed that EcNA@Cur‐Lip‐FA achieved stronger and more persistent intestinal colonization than EcNA alone. These findings underscored the potential of EcNA@Cur‐Lip‐FA as an integrated diagnostic and therapeutic platform for CRC, combining both enhanced drug delivery and tumor imaging in a single system.

### 2.2 Cellular Uptake, Cytotoxicity, and Antitumor Efficacy of EcNA@Cur‐Lip‐FA in Colorectal Cancer Cells

Based on the unique physicochemical properties of the integrated diagnostic and therapeutic platform EcNA@Cur‐Lip‐FA, this study systematically evaluated its cellular uptake efficiency, cytotoxicity, and antitumor efficacy in the CT26 CRC cell line. To monitor intracellular accumulation, we investigated the cellular uptake of Cy5.5‐labeled EcNA@Cur‐Lip‐FA by CT26 cells. After 1 h of co‐incubation, red fluorescence appeared in CT26 CRC cells, indicating the initial uptake of EcNA@Cur‐Lip‐FA by tumor cells. As the incubation time increased, the intracellular red fluorescence signal gradually intensified and became more pronounced at 8 h (Figure S9). The results demonstrated that EcNA@Cur‐Lip‐FA can be effectively taken up by tumor cells, thereby enabling effective antitumor activity.

**FIGURE 2:**
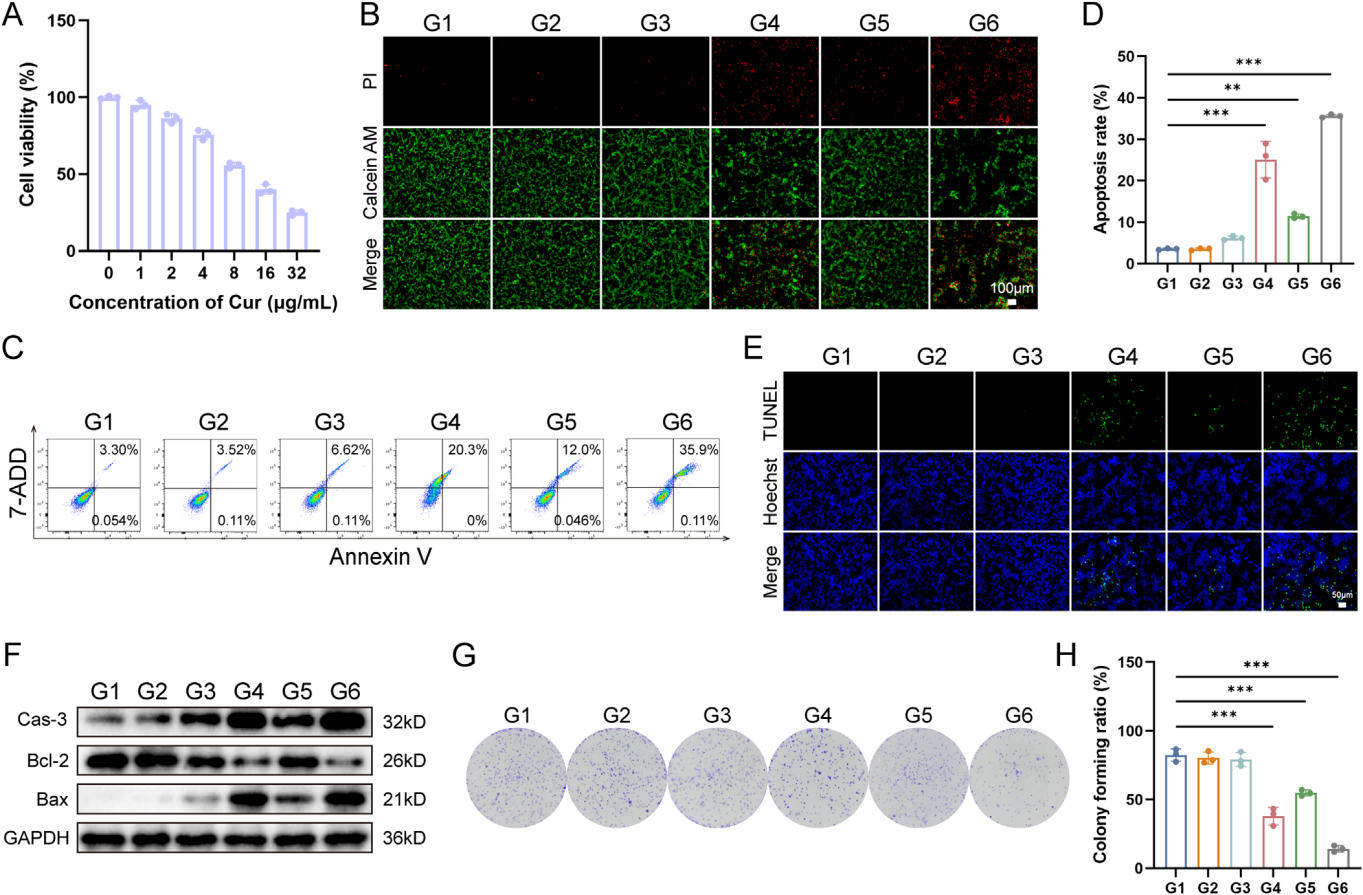
In vitro antitumor efficacy. (A) Cytotoxicity impact on CT26 cells (*n* = 4) after administration with different concentrations of EcNA@Cur‐Lip‐FA. (B) Fluorescence staining images of CT26 cells using Calcein‐AM/PI double staining method (*n* = 3, scale bar: 100 µm). (C) Annexin V‐FITC/PI double staining by FCM analysis and (D) corresponding statistical results (*n* = 3). (E) Fluorescence images of TUNEL apoptosis detection in CT26 cells (*n* = 3, scale bar: 50 µm). (F) WB assay of the expression levels of apoptosis‐related proteins. (G) Colony formation images of CT26 cells after treatments and (H) quantitative analysis results (*n* = 3). The statistical method adopted ANOVA analysis. Data are presented as the mean ± SD. *p < 0.05, **p < 0.01, and ***p < 0.001. Groups are designated to be G1: Control; G2: US; G3: EcNA; G4: Cur‐Lip‐FA + US; G5: EcNA@Cur‐Lip‐FA; G6: EcNA@Cur‐Lip‐FA + US.

To further investigate the antitumor effect of EcNA@Cur‐Lip‐FA, cell viability in CT26 cells was assessed using the CCK‐8 assay. As shown in Figure 2A, cell viability exhibited a dose‐dependent decrease with increasing concentration of EcNA@Cur‐Lip‐FA. We further calculated the half‐maximal inhibitory concentration (IC50) of EcNA@Cur‐Lip‐FA + US irradiation against CT26 cells, and used this concentration in the subsequent experiments. With the aim of evaluating the biosafety of EcNA@Cur‐Lip‐FA, we also performed CCK‐8 assays on human normal colonic epithelial NCM460 cells, and the results showed that EcNA@Cur‐Lip‐FA had low intrinsic cytotoxicity toward these normal cells (Figure S10). Subsequently, Calcein‐AM/PI double staining was used to visually assess cell viability. The results indicated that the EcNA@Cur‐Lip‐FA + US group showed the most dramatic shift in fluorescence signal compared to other groups (Figure 2B, Figure S11), mainly attributable to the synergistic effects of curcumin and SDT. Similarly, flow cytometry (FCM) demonstrated that the EcNA@Cur‐Lip‐FA + US group had an apoptosis rate of 35.6%, about 10 times higher than the control group and significantly higher than other groups (Figure 2C,D) (*p* < 0.001). And TUNEL assay (Figure 2E, Figure S12) confirmed DNA fragmentation, with a 17.7% TUNEL‐positive rate (*p* < 0.001). WB analysis (Figure 2F) indicated that in the EcNA@Cur‐Lip‐FA + US group, the expression levels of the apoptosis‐related proteins increased significantly, indicating the same result as mentioned above. Quantitative analysis results are presented in Figure S13.

Considering the confirmation that EcNA@Cur‐Lip‐FA can significantly induce cell death, we further explored its inhibitory effect on the proliferation of tumor cells. The colony formation assay (Figure 2G) revealed that EcNA@Cur‐Lip‐FA alone only caused a slight inhibitory effect. EcNA or US irradiation alone had limited effects on delaying colony formation. However, when EcNA or US irradiation was combined with Cur‐Lip‐FA, the inhibitory effect was substantially enhanced. Moreover, the EcNA@Cur‐Lip‐FA + US group (Figure 2H) demonstrated the most remarkable anti‐proliferative effect, with a colony formation rate of less than 20% (*p* < 0.001). The EdU assay (Figure S14) further confirmed this trend, showing that the number of EdU‐positive cells in the group treated with EcNA@Cur‐Lip‐FA + US was nearly undetectable. These results indicated that the treatment of EcNA@Cur‐Lip‐FA combined with US irradiation can achieve the optimal synergistic effect in terms of cytotoxicity against tumor cells and inhibition of tumor proliferation.

### 2.3 ROS‐Mediated DNA Damage and Immunogenic Cell Death Induced by EcNA@Cur‐Lip‐FA

**FIGURE 3:**
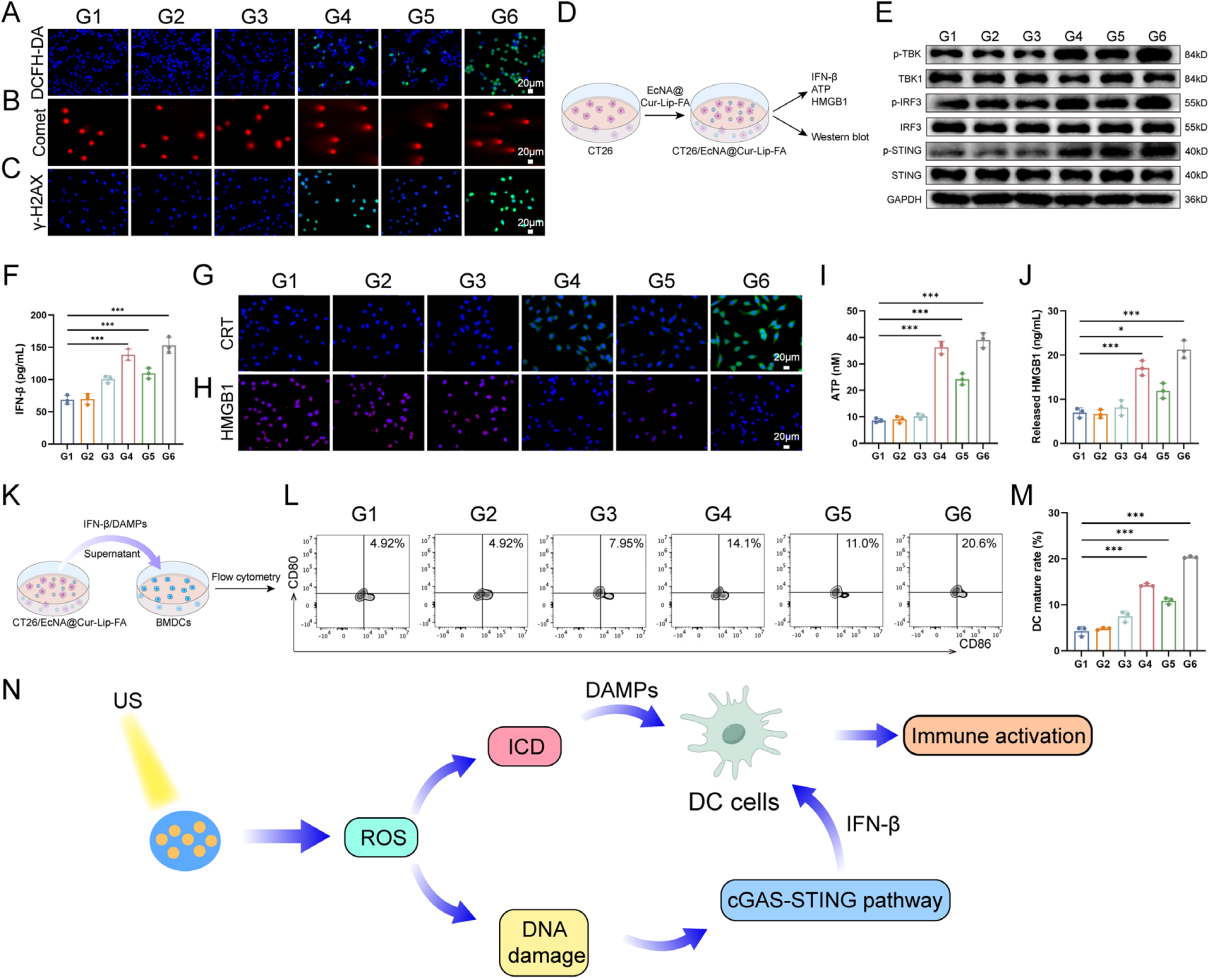
In vitro activation of the STING pathway and ICD effect. (A) Fluorescence images of ROS in CT26 cells after different treatments (*n* = 3, scale bar: 20 µm). (B) Comet assay images of CT26 cells after different administrations (*n* = 3, scale bar: 20 µm) and (C) fluorescence images of γ‐H2AX in CT26 cells after various treatments (*n* = 3, scale bar: 20 µm). (D) Schematic in vitro experimental procedure of the STING pathway activation and ICD effect in CT26 cells. (E) WB assay of the expression levels of proteins related to the cGAS‐STING pathway. (F) Expression level of IFN‐β. (G) CRT and (H) HMGB1 fluorescence images (*n* = 3, scale bar: 20 µm). (I) Extracellular ATP and (J) HMGB1 levels in CT26 cell supernatants (*n* = 3). (K) Scheme of activation of the cGAS‐STING pathway and ICD effect to promote DCs maturation. (L) FCM of BMDCs maturation after incubation with various drugs, and (M) proportion of mature DCs (*n* = 3). (N) Mechanism diagram of EcNA@Cur‐Lip‐FA + US irradiation activating the cGAS‐STING pathway and ICD effect, thereby inducing DCs maturation and enhancing antitumor immunity. Data are presented as the mean ± SD. *p < 0.05, **p < 0.01, and ***p < 0.001. The statistical method adopted ANOVA analysis. Groups are designated to be G1: Control; G2: US; G3: EcNA; G4: Cur‐Lip‐FA + US; G5: EcNA@Cur‐Lip‐FA; G6: EcNA@Cur‐Lip‐FA + US.

Encouraged by the satisfactory cytotoxic effects, we further explored the underlying antitumor mechanisms of EcNA@Cur‐Lip‐FA. Curcumin has been shown to exert antitumor effects by inducing ROS generation [24, 25, 26]. Thus, we detected ROS generation after different treatments. As shown in Figure S15A, the EcNA@Cur‐Lip‐FA + US irradiation group induced the highest level of ROS, which was 1.6 times that of Cur‐Lip‐FA + US irradiation and 2.7 times that of EcNA@Cur‐Lip‐FA (*p* < 0.001). The same trend was observed under fluorescence microscopy (Figure 3A, Figure S15B). Additionally, ROS are key mediators of cellular oxidative stress and their excessive accumulation can directly attack mitochondrial membranes [27, 28], causing a drop in mitochondrial membrane potential (MMP). Results showed (Figure S16) that after 24 h of treatment with EcNA@Cur‐Lip‐FA + US, red fluorescence decreased while green fluorescence increased significantly compared to the control group, which indicated a substantial decline in MMP.

Numerous studies have shown that ROS can induce DNA damage [29, 30]. To clarify the role of ROS‐mediated DNA damage in antitumor effects, we first used the comet assay to assess DNA single‐strand break or double‐strand break (DSB). As shown in Figure 3B, cells treated with EcNA@Cur‐Lip‐FA + US displayed distinct comet tails, indicating extensive ROS‐induced DNA DSBs. Similar to the comet assay, immunofluorescence analysis (Figure 3C, Figure S17) revealed a higher γ‐H2AX fluorescence intensity in the EcNA@Cur‐Lip‐FA + US group compared to the other groups. Since γ‐H2AX marks DNA DSBs [31], these results further confirmed that EcNA@Cur‐Lip‐FA with US irradiation primarily led to DNA DSBs. Additionally, the damaged DNA can be recognized by cGAS, which subsequently catalyzes the production of cGAMP, thereby activating the downstream STING‐TBK1‐IRF3 signaling pathway [32] (Figure 3D,E and Figure S18). And the activation of this pathway can trigger cells to secrete type I interferon (IFN‐β) (Figure 3F), which can further induce the maturation of DCs. In summary, these results indicated that EcNA@Cur‐Lip‐FA with US irradiation can induce ROS‐mediated DNA damage, increase cytoplasmic free dsDNA, and indirectly activate the cGAS‐STING pathway.

In addition to activating the cGAS‐STING pathway, numerous studies have demonstrated that curcumin induces ROS generation [24, 25, 26], causing endoplasmic reticulum stress and subsequently triggering immunogenic cell death (ICD), characterized by calreticulin (CRT) exposure on the cell surface and the release of damage‐associated molecular patterns (DAMPs), such as HMGB1 and ATP [33, 34]. Figure 3G and Figure S19 uncovered that in the control, US irradiation alone, and EcNA‐alone groups, CT26 cells had very low CRT fluorescence intensity on their surface. However, after cells were treated with EcNA@Cur‐Lip‐FA + US, CRT fluorescence intensity peaked, doubling that of the Cur‐Lip‐FA + US irradiation group. Conversely, HMGB1 fluorescence intensity decreased (Figure 3H, Figure S20), as HMGB1 is released outside the cell during ICD. WB results mirrored a consistent trend (Figure S21), showing increased CRT and HSP70 levels and decreased HMGB1 levels. Moreover, compared to EcNA@Cur‐Lip‐FA alone, when EcNA@Cur‐Lip‐FA was combined with US irradiation, it markedly increased ATP secretion and HMGB1 release (Figure 3I,J), demonstrating that the sonodynamic effect can further amplify the ICD effect.

Furthermore, the secretion of IFN‐β and the release of DAMPs triggered by ICD can induce the maturation of DCs, which in turn activates antitumor immunity [35, 36]. Therefore, we next co‐cultured bone marrow‐derived dendritic cells (BMDCs) with supernatants collected from CT26 cells subjected to different treatments for 48 h (Figure 3K). FCM analysis (Figure 3L,M) demonstrated that in the EcNA@Cur‐Lip‐FA + US group, the positive rate of DC maturation (CD80^+^CD86^+^) was significantly higher than other groups. In summary, EcNA@Cur‐Lip‐FA combined with US irradiation exerts its immunostimulatory effects through a dual mechanism involving ROS‐mediated DNA damage and ICD. On the one hand, the elevated ROS levels induce DNA damage, which subsequently activates the cGAS‐STING pathway, resulting in the production of IFN‐β. This enhanced antigen presentation and promoted the maturation of DCs. On the other hand, ROS also trigger ICD, leading to the release of DAMPs from dying tumor cells, which further stimulated DCs maturation. Together, these two pathways (Figure 3N) synergistically promoted a robust antitumor immune response, highlighting the therapeutic potential of EcNA@Cur‐Lip‐FA + US as a powerful immune activator.

### 2.4 Anti‐Angiogenic Effects of EcNA@Cur‐Lip‐FA With US Irradiation in the Tumor Microenvironment

**FIGURE 4:**
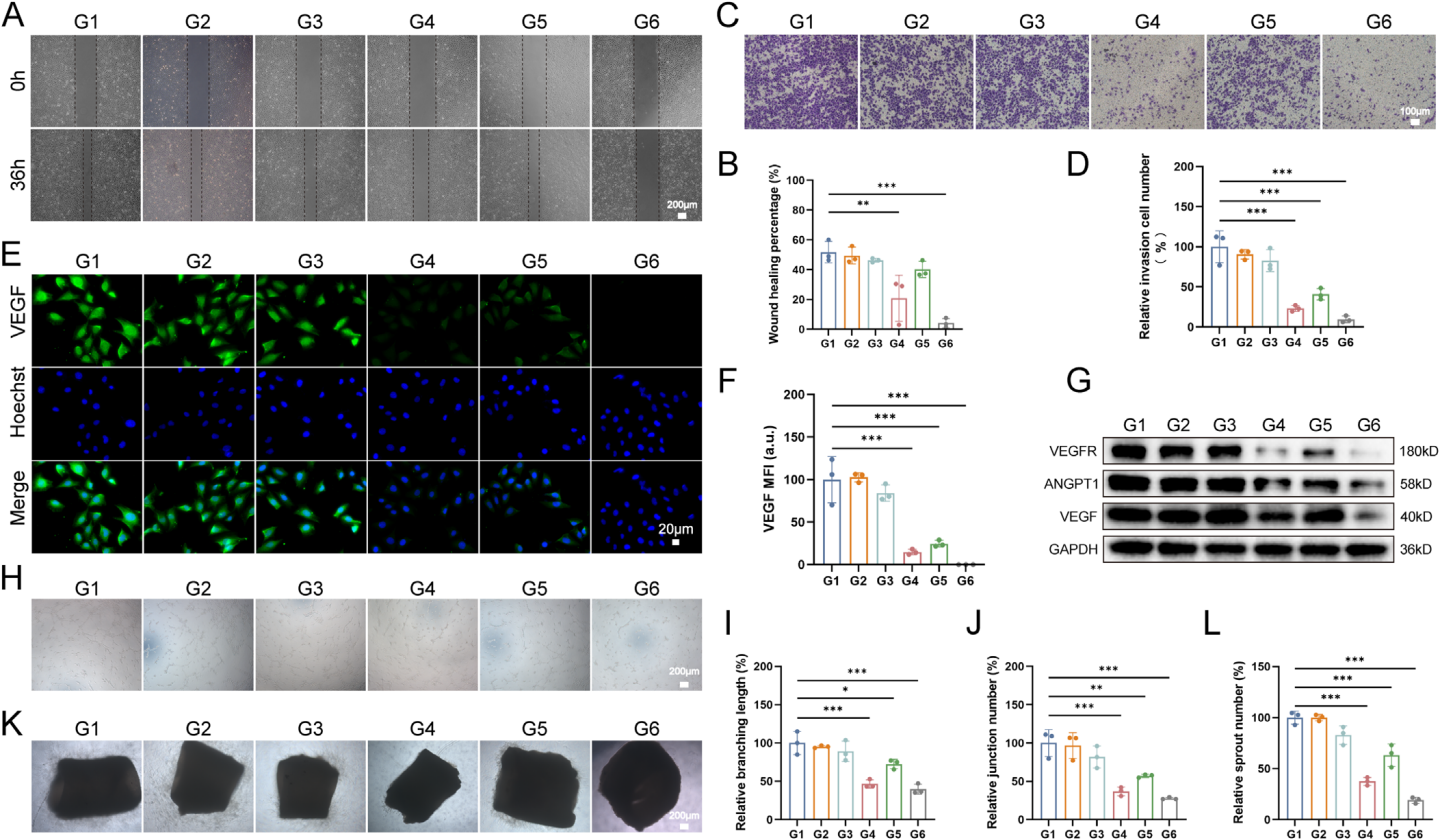
Anti‐angiogenic effects of EcNA@Cur‐Lip‐FA in vitro. (A) Scratch wound assay of HUVECs treated with different drugs at 0 h and 36 h (*n* = 3, scale bar: 200 µm) and (B) wound healing rate (*n* = 3). (C) Invasion images of HUVECs after different treatments (*n* = 3, scale bar: 100 µm) and (D) quantitative analysis results (*n* = 3). (E) Fluorescence images of VEGF in HUVECs after various treatments (*n* = 3, scale bar: 20 µm) and (F) corresponding quantitative results (*n* = 3). (G) WB analysis of the expression levels of angiogenesis‐related proteins. H) Tube formation ability of HUVECs under different interventions (*n* = 3, scale bar: 200 µm). (I) The tubule branch length (*n* = 3) and (J) the number of junctions (*n* = 3). (K) Representative optical microscopy images of aortic rings (*n* = 3, scale bar: 200 µm) and (L) the quantification of sprout number per ring (*n* = 3). The statistical method adopted ANOVA analysis. Data are presented as the mean ± SD. *p < 0.05, **p < 0.01, and ***p < 0.001. Groups are designated to be G1: Control; G2: US; G3: EcNA; G4: Cur‐Lip‐FA + US; G5: EcNA@Cur‐Lip‐FA; G6: EcNA@Cur‐Lip‐FA + US.

As mentioned above, EcNA@Cur‐Lip‐FA combined with US irradiation significantly enhances antitumor immunity through ROS‐induced ICD and activation of the cGAS‐STING signaling pathway. However, the immunosuppressive tumor microenvironment (ITM) is not only driven by immune cell dysfunction but also by hypoxia and nutrient deficiency resulting from abnormal tumor angiogenesis. Notably, curcumin, a natural compound with pleiotropic biological activities, has been shown to inhibit tumor angiogenesis [37]. To explore whether EcNA@Cur‐Lip‐FA + US irradiation could exert additional anti‐angiogenic effects, we conducted a series of angiogenesis‐related assays. A wound healing assay was performed to evaluate the effect of different treatments on HUVECs migration. As shown in Figure 4A, the scratch closure was significantly delayed after 36 h in the Cur‐Lip‐FA + US and EcNA@Cur‐Lip‐FA groups, especially in the EcNA@Cur‐Lip‐FA + US group. Quantitative analysis (Figure 4B) showed the wound healing rate dropped to just 4.16% (*p* < 0.001) in the EcNA@Cur‐Lip‐FA + US group, indicating a potent inhibitory effect on HUVECs migration. And transwell invasion assays (Figure 4C, D) revealed consistent results, with markedly reduced invasion capacity after EcNA@Cur‐Lip‐FA + US treatment. These findings demonstrated the synergistic efficacy of combined treatment in suppressing endothelial cell motility.

To further investigate the underlying mechanisms, immunofluorescence staining (Figure 4E,F) and WB analysis (Figure 4G, Figure S22) were employed. Results showed that the expression of key pro‐angiogenic markers, including VEGF, VEGFR1, and ANGPT1, was significantly downregulated following EcNA@Cur‐Lip‐FA + US treatment. The anti‐angiogenic effects were further validated through two classic in vitro assays. Moreover, the EcNA@Cur‐Lip‐FA + US group exhibited a 60.2% reduction in total branch length and a 72.2% decrease in node number compared to the control (*p* < 0.001), indicating severely impaired vascular network formation (Figure 4H–J). Similarly, the number of microvessel sprouts was significantly reduced by 80.9%, confirming strong inhibition of angiogenic sprouting (Figure 4K,L). Furthermore, tumor vascular normalization markedly ameliorated the hypoxic microenvironment and attenuated lactate accumulation (Figure S23). In summary, these results collectively indicate that EcNA@Cur‐Lip‐FA + US irradiation not only enhances immune activation but also effectively inhibits tumor angiogenesis by downregulating pro‐angiogenic factors and impairing HUVECs functions, thereby offering a dual therapeutic advantage.

### 2.5 Biosafety Evaluation of EcNA@Cur‐Lip‐FA: Hemocompatibility and Histopathological Assessment

Before evaluating the in vivo antitumor efficacy, we conducted extensive biosafety evaluations. As shown in Figure S24B, EcNA@Cur‐Lip‐FA, even at 320 µg/mL, did not trigger hemolysis, and the hemolysis rate was consistently under 5% across all concentrations, which met blood compatibility requirements for biomaterials. H&E staining of major organs (heart, liver, spleen, lung, kidney, and intestine) (Figure S24A) further confirmed no pathological abnormalities, such as cellular vacuolization, nuclear shrinkage, or inflammatory infiltration, in all groups. Moreover, after exposure to EcNA@Cur‐Lip‐FA, the relevant hematological and biochemical indicators in mice (Figure S24C) showed no significant variation from the control group. These outcomes offered dependable safety backing for the subsequent in vivo antitumor investigations of EcNA@Cur‐Lip‐FA.

### 2.6 Assessment of Therapeutic Capabilities of EcNA@Cur‐Lip‐FA in Orthotopic Colorectal Cancer Models

**FIGURE 5:**
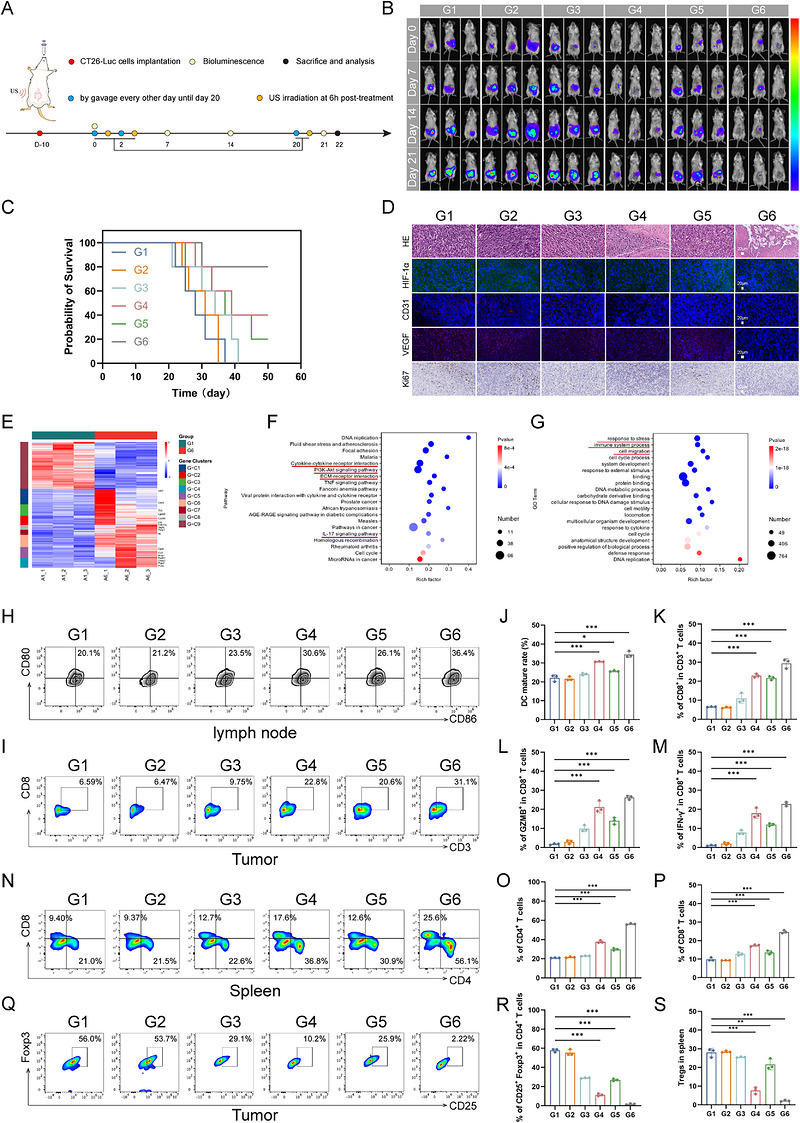
Evaluation of therapeutic efficacy against orthotopic tumors and effects on the tumor immune microenvironment. (A) Scheme of establishment and treatment of CT26 orthotopic tumor mouse model. (B) Bioluminescence imaging of tumors at different time points. (C) Survival curve of mice in different groups. (D) H&E staining, Ki67 staining, CD31 staining, HIF‐1α staining (scale bar: 50 µm), and VEGF staining (scale bar: 20 µm) of tumor tissues in each group. (E) Heatmap of differentially expressed genes in tumor tissue after treatment (*n* = 3). (F) KEGG enrichment analysis of the pathways involved in the biological effects induced by EcNA@Cur‐Lip‐FA + US irradiation (*n* = 3). (G) Upregulated biological process in tumor tissues based on GO annotation analysis (*n* = 3). (H) FCM of DCs in lymph nodes and (I) CD8^+^ T cells in tumor and quantitative analysis of (J) DCs and (K) CD8^+^ T cells. (L) The percentages of GZMB^+^ and (M) IFN‐γ in CD8^+^ T cells. (N) FCM of CD4^+^ T cells and CD8^+^ T cells in spleens and quantitative analysis of (O) CD4^+^ T cells and (P) CD8^+^ T cells. (Q) FCM and (R) quantitative data of Tregs in tumors after different treatments. (S) Quantitative data of Tregs in spleens. Data are presented as the mean ± SD. *p < 0.05, **p < 0.01, and ***p < 0.001. The statistical method adopted ANOVA analysis. Groups are designated to be G1: Control; G2: US; G3: EcNA; G4: Cur‐Lip‐FA + US; G5: EcNA@Cur‐Lip‐FA; G6: EcNA@Cur‐Lip‐FA + US.

Encouraged by the promising in vitro antitumor effects of EcNA@Cur‐Lip‐FA, we further explored its therapeutic efficacy in vivo. To this end, we established an orthotopic CT26 colon cancer model in BALB/c mice, which closely mimics the clinical and pathological features of human CRC. The treatment protocol is outlined in Figure 5A. US treatment was performed 6 h after gavage administration.

Tumor progression was dynamically monitored using in vivo bioluminescence imaging to assess treatment response (Figure 5B). The results revealed that both the control group and the US‐only group exhibited progressively increasing bioluminescence signals, indicating unchecked tumor growth and confirming that US irradiation alone had no therapeutic effect. In contrast, the Cur‐Lip‐FA + US and EcNA@Cur‐Lip‐FA groups showed modest tumor inhibition, reflected by relatively slower increases in bioluminescence intensity. Remarkably, the EcNA@Cur‐Lip‐FA + US group demonstrated nearly complete suppression of bioluminescence signals throughout the treatment period, suggesting a robust inhibition of tumor growth. This result not only highlights the synergistic therapeutic effect of EcNA@Cur‐Lip‐FA combined with US irradiation but also suggests enhanced lesion detectability and diagnostic accuracy. Importantly, no significant changes in body weight were observed across all groups during treatment (Figure S25), indicating that the formulation had minimal systemic toxicity. At the end of the experiment, the colorectum was excised and visually inspected. In the EcNA@Cur‐Lip‐FA + US group, no visible tumors, signs of intestinal obstruction, edema, peritoneal metastasis, and liver metastasis were observed (Figure S26). Furthermore, this group also exhibited a significantly prolonged survival time (Figure 5C). These findings collectively confirm the potent in vivo antitumor efficacy and safety of EcNA@Cur‐Lip‐FA combined with US irradiation, positioning it as a promising strategy for CRC treatment.

The results of H&E staining (Figure 5D) revealed apoptosis and necrosis of tumor cells. In contrast, other treatment groups exhibited varying degrees of nuclear enlargement, hyperchromasia, and increased mitotic figures. Immunofluorescence analysis (Figure 5D, Figure S27) further highlighted significant differences between the EcNA@Cur‐Lip‐FA + US group and the control group. Specifically, the EcNA@Cur‐Lip‐FA + US group showed a higher number of TUNEL‐positive cells (green fluorescence), indicating a marked increase in apoptosis (Figure S27). Additionally, the fluorescence intensity of HIF‐1α was significantly reduced in the EcNA@Cur‐Lip‐FA + US group compared to the control, suggesting an effective suppression of the tumor's hypoxic microenvironment. The reduced fluorescence intensities of CD31 and VEGF also indicated inhibition of angiogenesis. Moreover, Ki67, a nuclear protein marker associated with tumor cell proliferation and a key indicator of tumor malignancy, was notably lower in the EcNA@Cur‐Lip‐FA + US group. Collectively, these results suggested that EcNA@Cur‐Lip‐FA + US irradiation effectively inhibits tumor cell proliferation, induces apoptosis, alleviates the hypoxic tumor microenvironment, and exerts anti‐angiogenic effects, which are consistent with the findings from in vitro studies.

### 2.7 Modulation of Tumor Immune Microenvironment by EcNA@Cur‐Lip‐FA With Us Irradiation: Transcriptomic Profiling and Enhanced Immune Activation

To assess the impact of EcNA@Cur‐Lip‐FA combined with US irradiation on the ITM in vivo, we first performed transcriptomic analysis on tumor tissues from treated mice. The heatmap (Figure 5E) revealed significant differences in gene expression between the EcNA@Cur‐Lip‐FA + US group and the control group (*p* < 0.05), confirming a substantial alteration in the immune gene profile. The volcano plot (Figure S28) identified 983 upregulated genes and 583 downregulated genes (*p* < 0.05), providing a comprehensive overview of the treatment's effect on gene expression. The functional interaction network (Figure S29) further suggested that these differentially expressed genes are closely interconnected, which could amplify their biological effects. KEGG enrichment analysis (Figure 5F) highlighted key signaling pathways associated with immune response regulation, including cytokine‐cytokine receptor interactions, PI3K‐Akt signaling, and ECM‐receptor interactions (*p* < 0.05). Furthermore, GO enrichment analysis (Figure 5G) revealed that the upregulated genes were primarily involved in critical biological processes such as immune system activation, stress response, cell migration, cell cycle regulation, and protein binding (*p* < 0.05). Overall, these findings indicated that EcNA@Cur‐Lip‐FA combined with US irradiation significantly alters the expression of immune‐related genes in tumor tissues.

Given the in vitro immune activation capability of EcNA@Cur‐Lip‐FA and the results of transcriptomic analysis, we further investigated the in vivo systemic immune activation capability of EcNA@Cur‐Lip‐FA. DCs, as key antigen‐presenting cells (APCs), are essential for initiating T‐cell immune responses. Therefore, we evaluated the effects of different treatment groups on DCs maturation and antitumor immunity using FCM. Our results revealed that EcNA@Cur‐Lip‐FA combined with US irradiation could significantly enhance the proportion of mature CD80^+^CD86^+^ DCs in both tumor‐draining lymph nodes (Figure 5H,J) and tumor tissues (Figure S30), compared to the other groups. This suggested that EcNA@Cur‐Lip‐FA could effectively promote DCs maturation, which is crucial for antigen presentation and T cell activation. Furthermore, the EcNA@Cur‐Lip‐FA + US group exhibited a substantial increase in CD8^+^ cytotoxic T cells within the tumor (Figure 5I,K), showing an approximately 4.5‐fold increase compared to the control group. Additionally, there was a notable rise in the proportion of CD8^+^ T cells secreting granzyme B (Figure 5L, Figure S31) and IFN‐γ (Figure 5M, Figure S32), both of which are markers of enhanced cytotoxic activity. The EcNA@Cur‐Lip‐FA + US irradiation treatment also significantly increased the presence of both CD4^+^ and CD8^+^ T cells in the spleens (Figure 5N–P), suggesting systemic activation of T‐cell responses. Importantly, there was a significant reduction in the proportion of regulatory T cells (Tregs), which are known to suppress immune responses, in both the tumor (Figure 5Q,R) and spleens (Figure 5S, Figure S33). This decrease in Tregs indicated that the treatment effectively disrupts the tumor's immunosuppressive environment, promoting a more robust antitumor immune response. Additionally, a significant upregulation of pro‐inflammatory cytokines IL‐6, TNF‐α, and IL‐12p70 in serum was measured in the EcNA@Cur‐Lip‐FA + US group using enzyme‐linked immunosorbent assays (ELISA) (Figure S34A‐C). These cytokines contribute to creating an inflammatory microenvironment that supports the activation of the antitumor immune system. In conclusion, the combination of EcNA@Cur‐Lip‐FA and US irradiation effectively promotes DCs maturation and enhances the activation of CD8^+^ T cells, thereby reshaping the tumor immune microenvironment and producing a robust antitumor effect.

### 2.8 Inhibition of Liver Metastasis by EcNA@Cur‐Lip‐FA With Us Irradiation

**FIGURE 6:**
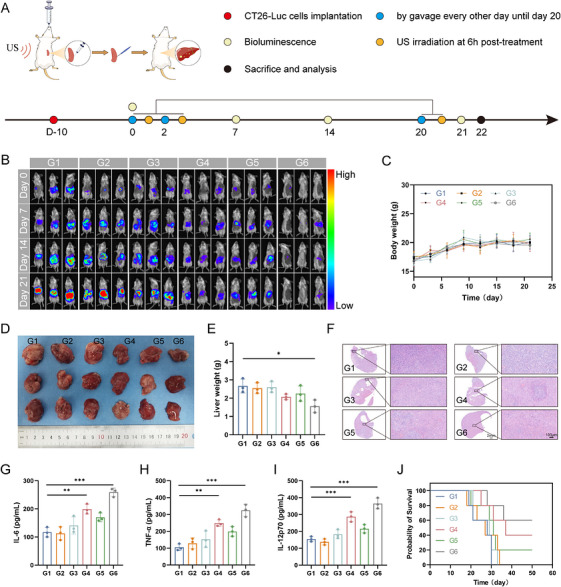
Evaluation of antitumor efficacy in the liver metastasis model. (A) Scheme of establishment and treatment of liver metastatic tumor model. (B) Bioluminescence imaging at different times. (C) Body weights of the CT26 liver metastasis tumor mice during the experimental period (*n* = 5). (D) Liver images and (E) liver organ weight at the end of the experiment (*n* = 3). (F) H&E staining was performed on liver tissues after different treatments (*n* = 3, scale bar: 2000 and 100 µm). Serum levels of (G) IL‐6, (H) TNF‐α, and (I) IL‐12p70. (J) Survival curve of mice in different groups. The statistical method adopted ANOVA analysis. Data are presented as the mean ± SD. *p < 0.05, **p < 0.01, and ***p < 0.001. Groups are designated to be G1: Control; G2: US; G3: EcNA; G4: Cur‐Lip‐FA + US; G5: EcNA@Cur‐Lip‐FA; G6: EcNA@Cur‐Lip‐FA + US.

Given the promising antitumor effect of EcNA@Cur‐Lip‐FA+US irradiation on primary tumors, we further investigated its impact on tumor metastasis. A liver metastasis model was established through splenic injection of CT26‐Luc cells, and the animals were treated according to the experimental protocol (Figure 6A). Throughout the treatment, bioluminescence signals were recorded using small animal in vivo imaging to monitor liver metastasis (Figure 6B). Body weight measurements taken every 3 days displayed no significant changes across groups (Figure 6C). Liver photographs (Figure 6D) revealed dense metastatic nodules on the liver surfaces in the PBS, US irradiation, and EcNA groups, with bulky metastases, hard texture, whitening, and almost complete loss of normal liver morphology. In contrast, the livers in the EcNA@Cur‐Lip‐FA + US irradiation group maintained good morphology, a bright red color, and fewer or no liver nodules. Additionally, the liver organ coefficient (Figure 6E) in the EcNA@Cur‐Lip‐FA group was reduced compared to the control group (*p* < 0.05). H&E staining further confirmed (Figure 6F) extensive infiltration of invasive tumor cells in the liver of the control group, whereas only scattered tiny lesions were observed in the EcNA@Cur‐Lip‐FA + US group, and some liver tissues showed almost no tumor metastasis. Interestingly, IL‐6, TNF‐α, and IL‐12p70 (Figure 6G–I) are crucial antitumor cytokines. Their levels rose significantly in mice subjected to EcNA@Cur‐Lip‐FA + US irradiation therapy. Finally, the survival prognosis curve showed that EcNA@Cur‐Lip‐FA + US irradiation significantly prolonged the survival of tumor‐bearing mice (Figure 6J). These evidence demonstrated that treatment with EcNA@Cur‐Lip‐FA + US dramatically suppressed CRC liver metastasis and significantly prolonged survival, underscoring its potential as a therapeutic strategy.

## 3 Discussion

CRC is one of the most common malignant tumors of the digestive tract [38]. Early accurate diagnosis and effective treatment are crucial for improving patient prognosis [39]. EcN, as one of the most widely applied engineered probiotics, demonstrates broad application prospects in the field of gastrointestinal diseases due to its outstanding biological characteristics. Additionally, as a facultative anaerobe, EcN can achieve targeted colonization in hypoxic microenvironments such as tumors or the colon, thereby serving as a precision drug delivery platform to enhance drug targeting and bioavailability [40, 41]. Notably, EcN is not only applicable for disease treatment but also for diagnosis [42]. By inducing EcN to express ARGs and administering them into the body, due to the significant acoustic impedance mismatch between these vesicles and the surrounding biological tissues, their presence significantly enhances the intensity of US backscatter. As a result, the contrast of diagnostic images is improved, and real‐time US imaging of their distribution within tumor tissues can be achieved, thereby facilitating accurate tumor diagnosis.

Additionally, to enhance the stability and efficacy of curcumin in vivo, researchers have developed various drug delivery platforms based on nano‐delivery systems [43, 44]. Liposomes, in particular, show promise for delivering curcumin, thanks to their unique phospholipid bilayer structure, which can accommodate both lipophilic and hydrophilic drugs [22, 45]. Moreover, by modifying the surface of liposomes with FA, which can actively target tumors through the ligand‐mediated mechanism, the uptake efficiency of liposomes in specific tumor cells can be further enhanced [46]. Furthermore, the formation of a protective layer is facilitated by poly (allylamine hydrochloride) (PAH), which establishes high‐density multivalent electrostatic adhesion and steric hindrance on the surfaces of both EcN cells and curcumin‐loaded liposomes. This, in turn, further supports the stability of NPs in the complex intestinal microenvironment. Under the influence of US, curcumin is activated, generating ROS‐driven ICD, which serve as potent danger signals to recruit and promote the maturation of dendritic cells (DCs), further activating anti‐tumor immunity.

**FIGURE 7:**
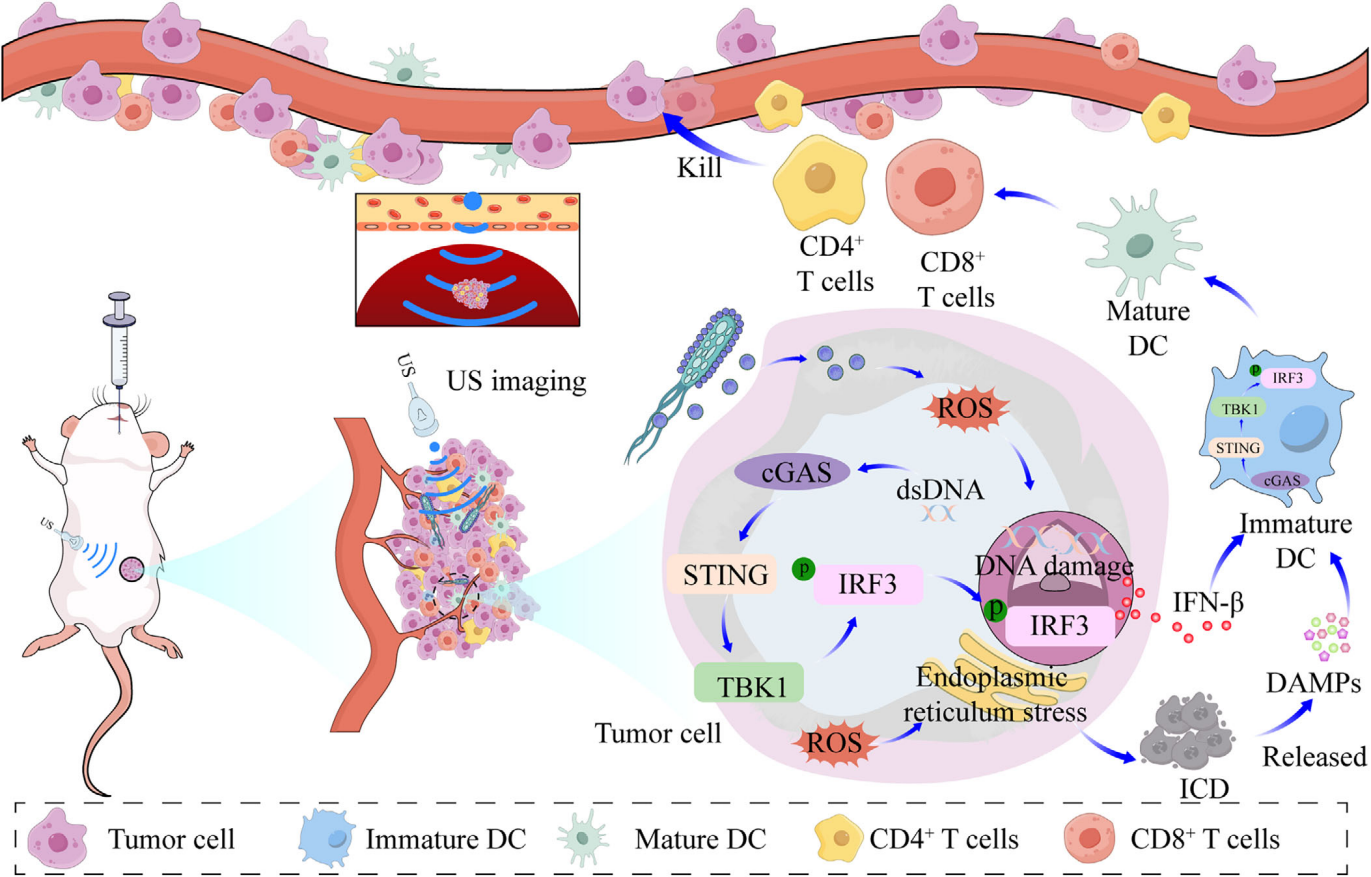
Schematic illustration of the EcNA@Cur‐Lip‐FA‐mediated ARGs expression and multidimensional immune activation in noninvasive imaging‐immunotherapy for CRC treatment.

In conclusion, we developed EcNA@Cur‐Lip‐FA, an antitumor nanosystem for CRC diagnosis and therapy (Figure 7). This nanosystem combines genetically engineered probiotic EcN, FA‐modified curcumin loaded liposomes. FA enables tumor‐specific accumulation, EcN modulates gut microbiota and boosts immunity, and US irradiation triggers cGAS‐STING activation, ICD induction, and anti‐angiogenesis via curcumin. In vitro and in vivo studies both confirmed potent inhibition of tumor growth, proliferation, and metastasis. Ultimately, this nanosystem enables real‐time, noninvasive diagnosis and synergistic therapy, offering a scalable paradigm for comprehensive cancer diagnosis and treatment.

## 4 Materials and Methods

### 4.1 Preparation of Products

The pET28a‐bARG1 plasmid was transformed into EcN, and the transformants were cultured in LB at 37°C to A_600_ 0.4–0.6, induced with IPTG at 30°C for 24 h, and harvested by centrifugal flotation (300× *g*, 4°C, 4 h) to obtain EcNA. For Cur‐Lip‐FA preparation, curcumin, DOPC, DOPG, DSPE‐PEG‐FA, and cholesterol (2:20:5:5:2.5 mg) were dissolved in chloroform, dried, hydrated with PBS, sonicated (130 W, 5 min, ice bath), and extruded through polycarbonate membranes to form uniform liposomes. Finally, EcNA (OD_600_ = 2.0, 900 µL) was incubated with PAH (100 µL) for 15 min, then reacted with Cur‐Lip‐FA (100 µL) at room temperature for 2 h; the product was collected by centrifugation, resuspended in PBS (4°C).

### 4.2 Colorectal Orthotopic Tumor Model

Five‐week‐old BALB/c mice (SPF Biotechnology) were housed at the Laboratory Animal Center of Guangxi Medical University. CT26‐Luc cells in log phase were mixed 1:1 with Matrigel to a final concentration of 5 × 10^6^ cells/50 µL. Under anesthesia, 50 µL of the suspension was injected obliquely into the cecal wall muscular layer using a 30‐G needle, and the wound was sutured. Mice were divided into six groups (*n* = 5): (1) Control (untreated), (2) US alone (1.0 MHz, 50% duty cycle, 2.0 W/cm^2^, 5 min), (3) EcNA (1 × 10^10^ CFU/kg), (4) Cur‐Lip‐FA (Cur: 100 mg/kg) + US (same parameters), (5) EcNA@Cur‐Lip‐FA (Cur: 100 mg/kg), and (6) EcNA@Cur‐Lip‐FA (Cur: 100 mg/kg) + US. Body weight and tumor growth (via in vivo imaging) were monitored regularly.

### 4.3 In Vitro Cytotoxicity Assay

CT26 and NCM460 cells were seeded at 10^4^ cells/well in 96‐well plates and cultured overnight at 37°C with 5% CO_2_. After treatment with various concentrations of EcNA@Cur‐Lip‐FA for 8 h, cells were exposed to US (1.0 MHz, 50% duty cycle, 1.0 W/cm^2^) for 5 min, then incubated for another 16 h. CCK‐8 solution (ZP328‐3, Zomanbio, China) was added and the absorbance was read at 450 nm.

### 4.4 Western Blotting

Cells were initially washed twice with PBS and subsequently lysed with NP‐40 (N8032, Solarbio, China) for 30 min on ice. The cell mixture was then centrifuged at 15,000 *g* for 15 min at 4°C. Protein concentrations were determined using a BCA protein assay kit (P0012S, Beyotime, China). Equal amounts of protein were loaded onto the SDS‐PAGE gel (PG112, Epizyme, China). Following electrophoresis, the proteins were transferred to a PVDF membrane. Subsequently, the membrane was incubated with primary antibodies, followed by secondary antibodies. Primary antibodies included Cas‐3 (ABclonal, 1:1000), Bcl‐2 (ABclonal, 1:1000), Bax (ABclonal, 1:1000), GAPDH (Abways, 1:1000), p‐TBK1 (ABclonal, 1:1000), TBK1 (ABclonal, 1:1000), p‐IRF3 (ABclonal, 1:1000), IRF3 (ABclonal, 1:1000), p‐STING (AffInity, 1:1000), STING (ABclonal, 1:1000), HSP70 (ABclonal, 1:1000), Calreticulin (ABclonal, 1:1000), HMGB1 (ABclonal, 1:1000), VEGFR (Abways, 1:1000), ANGPT1 (ABclonal, 1:1000), VEGF (ABclonal, 1:1000), FOLR1 (STARTER, 1:1000), and β‐tubulin (ABclonal, 1:1000). The anti‐rabbit IgG antibody (ABclonal, 1:1000) and the anti‐mouse IgG antibody (ABclonal, 1:1000) were used as the secondary antibody. Images were viewed using the Image Lab Software (V.6.0.1; Bio‐Rad).

### 4.5 Tube Formation Assay

Before the experiment, the matrix gel was applied to the 96‐well plate. Then, human umbilical vein endothelial cells (HUVECs) that had undergone different treatments were taken and their density was adjusted to 3 × 10^5^ cells/mL. Next, 100 µL of the cell suspension was vertically dropped onto the surface of the solidified Matrigel (354248, Corning Incorporated, USA) and the plate was left to stand horizontally for 5 min.

### 4.6 Flow Cytometry

The tumor, spleens, and lymph nodes were harvested and cut into small pieces at the end of experiment. Then, these pieces were then dissociated into single‐cell suspensions. Next, the single cells were stained with fluorescein‐labeled antibodies:Zombie‐APC‐CY7, CD86‐BV421, CD8‐FITC, GZMB‐BV421, CD3‐PE‐CY7, F4/80‐PC5.5, CD4‐APC, CD25‐BV421, Foxp3‐PE, and IFN‐γ‐PE, following the manufacturer's protocols from Biolegend, USA. Eventually, the stained cell suspensions were loaded onto a flow cytometer for detailed analysis.

## 5 Statistical Analysis

All data were presented as the mean ± standard deviation (SD). Statistical significance was determined using ANOVA. *p* values less than 0.05 were considered statistically significant, and *p < 0.05, **p < 0.01, ***p < 0.001. All statistical analyses were performed using GraphPad Prism software version 9.0.

## Author Contributions

Yunxi Huang, Kang Zhang, and Weizhong Tang designed and supported the project. Jie Long, Yunfang Yu, Tongrui Shang, Xuezhong Mo, Feng Lin, Yunxi Huang, Zixuan Liang, and Renchuan Liang performed the experiments and collected the data. Jie Long, Feng Lin, Olivia Monteiro, Daniel Baptista‐Hon, and Man Tong analyzed and interpreted the data. Jie Long, Yunxi Huang, and Yunfang Yu wrote, reviewed, and edited the paper. Tongrui Shang, Dominic Chi‐Chung FOO, and Lui Ng provided constructive comments on the manuscript revision. All authors have read and approved the contents of this article.

## Funding

This work was financially supported by China Postdoctoral Science Foundation (Grant Number MD763947), Science and Technology Base and Talent Special Project of Guangxi (Grant Number GuikeAD24010010), the Macau Science and Technology Development Fund, Macau (Grant Number 0003/2021/AKP), and Guangdong Science and Technology Department (No. 2024B1212030002).

## Ethics Statement

All experimental procedures were approved by the Ethics Committee of Guangxi Medical University Cancer Hospital (Nanning, China) with an approval number: LW2025040. All animal care and procedures were performed according to the institutional guidelines.

## Conflicts of Interest

Professor Kang Zhang serves as an Associate Editor of MedComm. However, he did not participate in the peer review process or editorial decision‐making for this manuscript. All other authors declare no conflicts of interest.

## Supporting information




**Supporting File 1**: mco270871‐sup‐0001‐SuppMat.docx

## Data Availability

The data that support the findings of this study are available from the corresponding author upon reasonable request.
