# Supplementary material for: Genetically Engineered Probiotic Nanoplatform for Ultrasound‐Enhanced Colorectal Cancer Therapy via Targeted Delivery, Immune Modulation, and Anti‐Angiogenesis
Source: MedComm (2020). 2026 Jul 16;7(8):e70871. doi: 10.1002/mco2.70871 (PMC13376840; doi:10.1002/mco2.70871)
Supplement: Supplementary file 1 — Supporting File 1: mco270871‐sup‐0001‐SuppMat.docx [file MCO2-7-e70871-s001.docx]

Supporting Information

**Genetically Engineered Probiotic Nanoplatform for Ultrasound-Enhanced Colorectal Cancer Therapy via Targeted Delivery, Immune Modulation, and Anti-Angiogenesis**

**Jie Long^1†^**, **Tongrui Shang^2†^**, **Xuezhong Mo^1^**^†^, **Yunfang Yu,^2,3,4,5^**^†^, **Feng Lin^1^**, **Zixuan Liang^1^**, **Olivia Monteiro^4,6^**, **Daniel Baptista-Hon^4,6^**, **Dominic Chi-Chung FOO^7^**, **Lui Ng^7^**, **Renchuan Liang^1^**, **Man Tong^8^**, **Weizhong Tang^1,^***, **Kang Zhang^3,9,10,11,^***, **Yunxi Huang^1,^***

^1^Department of Otolaryngology-Head and Neck Surgery, Colorectal and Anal Disease Unit, Department of Gastrointestinal Surgery, Guangxi Medical University Cancer Hospital, Guangxi Key Laboratory of Basic and Translational Research for Colorectal Cancer, No.71 Hedi Road, Nanning, Guangxi Zhuang Autonomous Region, China.

^2^Guangdong Provincial Key Laboratory of Malignant Tumor Epigenetics and Gene Regulation, Guangdong-Hong Kong Joint Laboratory for RNA Medicine, Sun Yat-sen Memorial Hospital, Sun Yat-sen University, Guangzhou, China.

^3^Macau Institute for AI in Medicine and Faculty of Medicine, Macau University of Science and Technology, Taipa, Macao, China.

^4^Guangdong Provincial Key Laboratory of Cancer Pathogenesis and Precision Diagnosis and Treatment, AI Big Data Laboratory, Shenshan Medical Center, Memorial Hospital of Sun Yat-sen University, Shanwei, China.

^5^The First Affiliated Hospital, Jinan University, Guangzhou, China

^6^School of Medicine, University of Dundee, Dundee, UK.

^7^Department of Surgery, University of Hong Kong, Macao, China.

^8^School of Biomedical Sciences, The Chinese University of Hong Kong, Hong Kong SAR, China.

^9^State Key Laboratory of Eye Health, Eye Hospital and Institute for Advanced Study on Eye Health and Diseases, Wenzhou Medical University, Wenzhou, China.

^10^National Clinical Research Center for Ocular Diseases, Eye Hospital, Wenzhou Medical University, Wenzhou, China.

^11^Guangzhou National Laboratory, Guangzhou, China.

**^†^**These authors contributed equally and considered co-first authors.

***Correspondence**

Weizhong Tang[,](mailto:tangweizhong@gxmu.edu.cn,) Email: tangweizhong@gxmu.edu.cn

and

Kang Zhang, Email: kang.zhang@gmail.com

and

Yunxi Huang, Email: [hyunxi163@163.com](mailto:hyunxi163@163.com)

**Experimental Section**

**Materials and Methods**

**1 Experimental Animal Model**

Colorectal Cancer Liver Metastasis Model: Five-week-old BALB/c mice were selected. Before inoculation, CT26-Luc cells in the logarithmic growth phase were prepared into a cell suspension, and the concentration was adjusted to 5×10^6^ cells/50 μL. After the mice were anesthetized, a 1.0 cm oblique incision was made below the left costal margin to expose the spleen. A 30G insulin syringe was used to slowly inject 50 μL of the cell suspension into the spleen, and the needle was left in place for 30 seconds before withdrawal. The muscle layer and skin were sutured in layers after the operation. The mice were divided into 6 groups(n=5): (1) Control, (2) US irradiation(1.0 MHz, 50% duty cycle, 2.0 W/cm^2^, 5 min), (3) EcNA (1×10^10^ CFU/kg), (4) Cur-Lip-FA(Cur: 100mg/kg)+US irradiation(1.0 MHz, 50% duty cycle, 2.0 W/cm^2^, 5 min), (5) EcNA@Cur-Lip-FA (Cur: 100mg/kg) and (6) EcNA@Cur-Lip-FA (Cur: 100mg/kg)+US irradiation (1.0 MHz, 50% duty cycle, 2.0 W/cm^2^, 5 min). The bioluminescence signal was monitored once a week using an in vivo imaging system in the course of the experiment, focusing on the dynamic changes of the splenic primary lesion and liver metastasis. At the end of the experiment, the weight of the liver was measured, and the pathological changes were confirmed by H&E staining.

BALB/c mice bearing CT26 cecal-wall orthotopic tumors received a single oral gavage of either EcNA@Cur-Lip-FA-Cy5.5 (1×10^10^ CFU/kg) or free Cy5.5 (equivalent dye dose). At 0, 3, 6, 12, 24 and 48 h post-administration, mice were anaesthetised and whole-body fluorescence was captured (IVIS Spectrum).

BALB/c mice bearing tumors were randomized to receive EcNA@Cur-Lip-FA-Cy5.5 or EcNA-Cy5.5 by oral gavage. At 3, 6, 12, 24 and 48 h, mice were euthanized; intact cecum and colon were gently rinsed to remove luminal content and immediately imaged (IVIS Spectrum).

**2 Transmission Electron Microscope Images**

Microscopic structures of bacteria and liposomes were observed by transmission electron microscopy (TEM). Before conducting TEM analysis, the samples were first dispersed in ultrapure water, then a drop of the sample was deposited onto a carbon-coated copper grid, and subsequently air-dried. For samples requiring negative staining, the copper grid was negatively stained with a 2% phosphotungstic acid solution and then air-dried.

**3 Atomic Force Microscope (AFM) Images**

The experiment adopted the tapping mode and was conducted on an atomic force microscope. The samples were dispersed in ultrapure water and then dropped onto the surface of mica. After they naturally dried, atomic force microscope detection was carried out.

**4 Co-localization Analysis**

The Cur-Lip-FA was stained with a lipophilic dye (DiO) and the bacteria were stained with DAPI for 10 minutes to to label bacterial DNA. Then, under a laser confocal microscope, fluorescence imaging of the cell nuclei and liposomal curcumin was achieved.

**5 Dynamic Light Scattering (DLS) and Zeta Potential Analysis**

The particle size of the prepared nanomedicine was measured by DLS. In addition, the zeta potential of the samples was measured using Zetasizer Nano ZS90 instrument (Malvern Instruments, UK).

**6 Drug Release Experiment**

Briefly, we first placed 0.2 mL of Cur-Lip-FA and EcNA@Cur-Lip-FA in a dialysis tube with a molecular weight cut-off of 8000. The experiment used PBS (pH5.0 and pH7.4) as the release medium to simulate the physiological conditions in the body. Samples of the release medium were taken at preset time points (0, 1, 2, 4, 8, 12, 16, 24 and 48 hours), and the content of curcumin in the samples was determined by UV-Vis spectrophotometry. By recording the cumulative release amount of curcumin at different time points, the release kinetic parameters can be calculated to evaluate the release characteristics of curcumin from samples.

**7 Cellular Uptake Behavior of EcNA@Cur-Lip-FA**

CT26 cells were seeded into 96-well plates at a density of 10^4^ cells per well and cultured for 12 hours. Subsequently, Cy5.5-modified EcNA@Cur-Lip-FA were added and co-incubated with the cells for 0, 1, 2, 4, and 8 hours. After three washes with PBS, Hoechst 33342 (C0031, Solarbio, China) was applied to stain the nuclei for 10 minutes at 37℃. Fluorescence imaging was subsequently performed using a fluorescence microscope.

**8 Live/Dead, ROS and JC-1 Assay**

After the CT26 cells attached to the bottom of the wells, they were subjected to various treatment conditions for 24 hours ((1) Control, (2) US irradiation(1.0 MHz, 50% duty cycle, 1.0 W/cm^2^, 5 min), (3) EcNA (1×10^6^CFU/mL), (4) Cur-Lip-FA(cur: 10 µg/mL)+US irradiation (1.0 MHz, 50% duty cycle, 1.0 W/cm^2^, 5 min), (5) EcNA@Cur-Lip-FA (cur: 10 µg/mL) and (6) EcNA@Cur-Lip-FA (cur: 10 µg/mL) + US irradiation (1.0 MHz, 50% duty cycle, 1.0 W/cm^2^, 5 min)). After 8 hours, for the groups requiring US irradiation, the cells were removed from the incubator and exposed to US irradiation with specific parameters and then returned to the incubator. Subsequently, the cells were stained with DCFH-DA (S0033S, Beyotime, China), JC-1 (C2006, Beyotime, China) and Calcein/PI (C2015S, Beyotime, China) probe in accordance with the instructions provided by the respective detection kit.

**9 Immunofluorescence Analysis**

CT26 cells were seeded into a 48-well plate at a density of 2×10^4^ cells per well and the treatment of cells was the same as described previously. Briefly, the cells were first fixed with 4% paraformaldehyde for 15 minutes, permeabilized for 10 minutes, and blocked at 37℃ for 30 minutes. The cells were incubated with primary antibody H2AX (ABclonal, 1:1000), Calreticulin (ABclonal, 1:1000), HMGB1 (ABclonal, 1:1000) and VEGF (ABclonal, 1:1000) at 4℃ overnight, and the fluorescent secondary antibody was incubated in a dark place for 1 hour. The nuclei were stained with Hoechst33342 (C0031, Solarbio, China) for 15min and then images were acquired by fluorescence microscope.

**10 Colony Formation Assay**

CT26 cells subjected to different treatments were seeded into 6-well plates at a concentration of 1000 cells per well, and cultured until visible colonies formed. Cells were washed twice with PBS, fixed with paraformaldehyde for 30 minutes, and stained with a 0.1% crystal violet solution (G1064, Solarbio, China). The number of colony-forming cells was counted using Image J.

**11 Annexin V/7-AAD/PI Assay**

CT26 cells were seeded into 6-well plates and incubated overnight at 37°C. Then, they were co-cultured with different experimental groups for 8 hours. Subsequently, the cells were subjected to or without US irradiation treatment (1.0 MHz, 50% duty cycle, 1.0 W/cm², 5 min). After an additional 16-hour incubation period, the ratios of apoptosis in each group were analyzed by flow cytometry using the Annexin V-APC/7-AAD /PI Apoptosis Detection Kit following the manufacturer's instructions (AT105, liankebio, China).

**12 TUNEL Staining and Cell Proliferation Assay**

Cells were treated as described above. Subsequently, the cells were subjected to fixation and permeabilization procedures, followed by TUNEL staining (C1086, Beyotime, China) for the detection of apoptosis and EdU staining (C10310-1, Ribobio, China) for the assessment of proliferation. Fluorescence microscopy was used to capture images.

**13 Comet Assay**

Initially, the cell suspension was combined with low-melting-point agarose and uniformly spread onto a microscope slide that had been pre-coated with normal-melting-point agarose. The mixture was then allowed to solidify. Subsequently, the slide was immersed in a lysis solution to remove cellular components, leaving only the DNA. Following this, the slide was placed into an electrophoresis chamber and subjected to electrophoresis at a low voltage. During this process, damaged DNA fragments migrated out of the nucleus, forming a characteristic “comet” tail. After completion of electrophoresis, the DNA was stained with a PI dye (KGA1302-20, KeyGEN, China). The resulting “comet” structures were then observed and analyzed under a fluorescence microscope to evaluate the extent of DNA damage.

**14 Detection of extracellular ATP Level**

CT26 cells were seeded in 6-well plates and treated in the same manner as previously described. After that, the supernatant was immediately collected for the detection of ATP levels in vitro using ATP assay kits (S0027, Beyotime, China).

**15 Isolation of BMDCs**

Under sterile conditions, the femurs and tibias of mice were removed. After cutting open the bones, bone marrow cells were flushed into RPMI1640 medium using a syringe to create a single - cell suspension. Subsequently, density gradient centrifugation was performed to isolate the low - density bone marrow cell population. The isolated bone marrow cells were cultured in RPMI1640 medium containing recombinant mouse GM - CSF (20 ng/mL). Regular semi - medium changes were carried out to supplement the cytokines. After 6 days, the cells were replated for further experiments.

**16 ELISA Analysis**

At the end of the experiment, the supernatant from the tumor cell cultures, as well as the blood from the mice were collected. An enzyme-linked immunosorbent assay (ELISA) was conducted following standard protocols to detect various cytokines, including HMGB1 (PH406, Beyotime), IFN-β (EK2236, Liankebio, China), IL-6 (EK206, Liankebio, China), IL-12p70 (EK212, Liankebio, China), and TNF-α (EMC102a, Neobioscience, China).

**17 Wound Scratch Assay**

Briefly, horizontal lines were drawn on the reverse side of the 6-well plate using a marker pen. After the cells covered the entire culture surface, Multiple straight scratch lines were made on the cell using a pipette tip. The wells were then washed once with PBS and serum-free medium was added for culture. cell migration was observed at 0 and 36 hours under microscope to record the scratch width. Finally, ImageJ software was used for statistical analysis according to the following formula: Scratch migration rate = (scratch width at 0h - scratch width at 36h) / scratch width at 0h × 100%.

**18 Invasion Assay**

After a 24-hour treatment with various treatment groups, the cells were digested. Invasion assays were conducted with transwell chambers (CLS3420, Corning Incorporated, USA). For the invasion assay, Matrigel (354248, Corning Incorporated, USA) was coated onto the chamber, and cells in serum-free medium were cultured on top. Medium containing 10% FBS was placed at the bottom of the chamber. Cells on the bottom surface of the membrane were fixed and stained with crystal violet after 24 hours.

**19 Aortic Ring Assay**

Rats aged 8 to 10 weeks were selected. After being euthanized, the abdominal aorta was completely dissected out, Subsequently, the lumen was rinsed three times with sterile PBS to remove residual blood. The aorta was then cut into 2-mm segments using sterile scissors. Before the experiment, Matrigel (354248, Corning Incorporated, USA) was pre-cooled to 4°C and spread at the bottom of a 96-well plate. The aortic rings were vertically embedded in the center of the matrix gel and covered with a second layer of matrix gel. Finally, 100 μL of serum-containing medium was added and the plate was placed in incubator. Eventually, the images were observed using a microscope.

**20 Biocompatibility and Biosafety of Nanoparticles**

To assess the biocompatibility and biosafety of nanoparticles, healthy female BALB/c mice aged 5 weeks were selected for different treatments via oral gavage. The mice were divided into six groups: (1) Control, (2) US irradiation (1.0 MHz, 50% duty cycle, 2.0 W/cm^2^, 5 min), (3) EcNA (1×10^10^ CFU/kg), (4) Cur-Lip-FA (Cur: 100mg/kg)+US irradiation (1.0 MHz, 50% duty cycle, 2.0 W/cm^2^, 5 min), (5) EcNA@Cur-Lip-FA (Cur: 100mg/kg) and (6) EcNA@Cur-Lip-FA (Cur: 100mg/kg) + US irradiation (1.0 MHz, 50% duty cycle, 2.0 W/cm^2^, 5 min). Treatment was administered every other day for 11 days, with a total observation period of 30 days. Subsequently, the major organs of the mice (heart, liver, spleen, lung, kidney, and intestine) were collected for H&E staining analysis. Additionally, venous blood was drawn to measure levels of red-blood-cell (RBC), white blood cell (WBC), platelet (PLT), lactate dehydrogenase (LDH), blood urea nitrogen (BUN), aspartate aminotransferase (AST), alanine aminotransferase (ALT) and creatinine (CREA).

**21 Hematoxylin and eosin(H&E) Staining**

The tumor and liver were fixed with paraformaldehyde, paraffin sections were made after pruning, dehydration and transparency, and the prepared paraffin sections were dewaxed, stained with hematoxylin (C0105S-1, Beyotime, China) dye for 10 minutes, and the sections were washed with running water for 5 minutes. The rinsed tissue sections were placed in 1% hydrochloric acid ethanol, decolored for 5-10 seconds, and rinsed with running water for 5 minutes. Then, the 1% ammonia solution (Acmec, China) was returned to blue for 5 minutes, and the running water was rinsed for 5 minutes. Subsequently, Tissue sections were placed in a prepared 0.5 % eosin dye (C0105S-2, Beyotime, China), stained at room temperature for 1-3 minute, and then decolored in 70%, 80%, and 95% ethanol successively, 5-10 seconds each time, and finally dehydrated twice with anhydrous ethanol. Xylene (534056, Merck, USA) was used to make the sections transparent and observed under the microscope after being sealed with neutral gum.

**22 TUNEL, HIF-1α, CD31 and VEGF Staining**

To evaluate apoptosis and the expression levels of Ki-67, HIF-1α, CD31 and VEGF in tumor tissues, paraffin sections were dewaxed, rehydrated and subjected to antigen retrieval (G1219-1L, Servicebio, China). For apoptosis detection, the same sections were then fixed and permeabilized according to the TUNEL kit protocol (ab66108, Abcam, UK) before terminal transferase labeling, followed by routine immunohistochemical staining for the remaining markers. Then, they were incubated with primary antibodies including Ki-67 (Servicebio, 1:1000), HIF-1α (Affinity, 1:1000), CD31 (Abclonal, 1:1000), VEGF (Abclonal, 1:1000) and then were incubated with secondary antibodies successively. Subsequently, a DAB chromogenic reagent was used for the chromogenic reaction (about 5-10 minutes), and the cell nuclei were counterstained with hematoxylin (C0105S-1, Beyotime, China). After dehydration through a gradient of ethanol, the sections were mounted with neutral gum (C0173-100ml, Beyotime, China) and the staining results of the tumor sections were observed under a microscope. TUNEL staining was performed to detect DNA fragmentation during apoptosis. Ki-67 staining reflected the rate of growth and proliferation of tumor cells. HIF-1α staining was used to assess cellular hypoxia. CD31 and VEGF staining was employed to evaluate tumor angiogenesis.

**Supplementary figures**


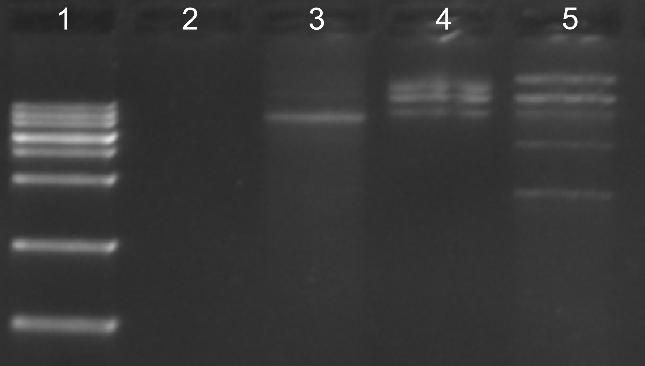


**Figure S1.** Gel electrophoresis. (Lane 1: marker. Lane 2: EcN. Lane 3: EcN transformed with ARGs. Lane 4: pure ARGs plasmid. Lane 5: plasmid from DH5α transformed with ARGs.)


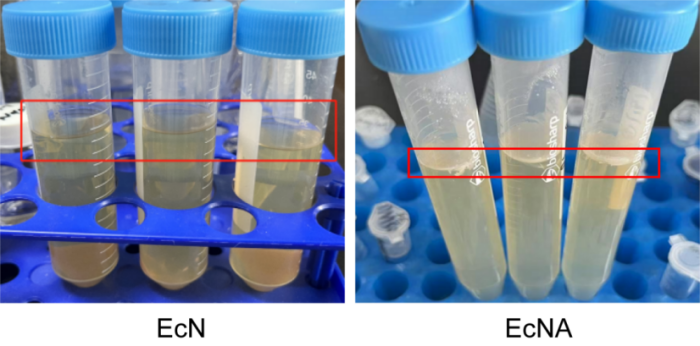


**Figure S2.** Pictures of the product after centrifugation.

**
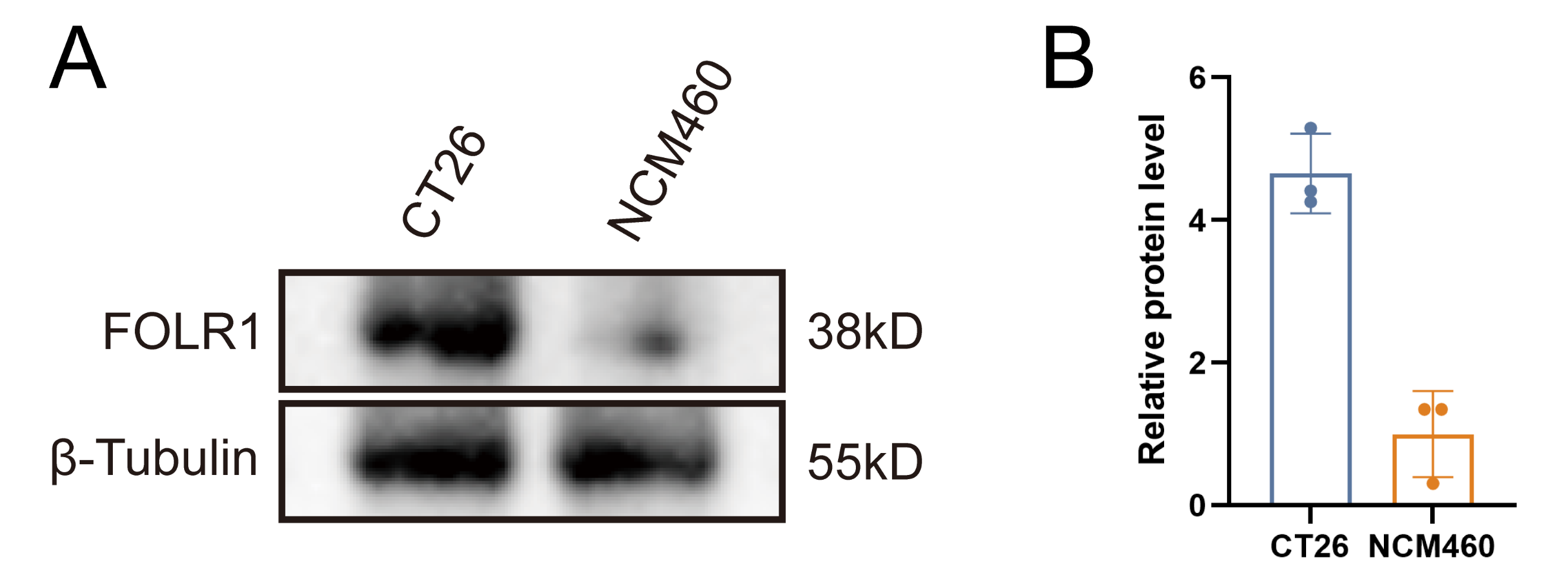
**

**Figure S3.** A) WB analysis of the expression levels of FOLR1 proteins and B) corresponding quantitative result. Data were shown as the mean ± SD.


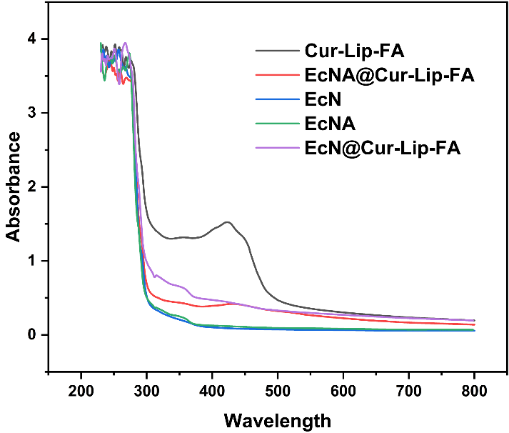


**Figure S4.** UV-vis spectra of different nanoparticles.


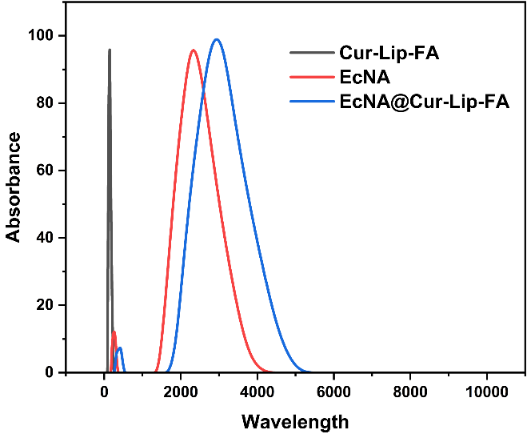


**Figure S5.** The particle size distribution detected by DLS.


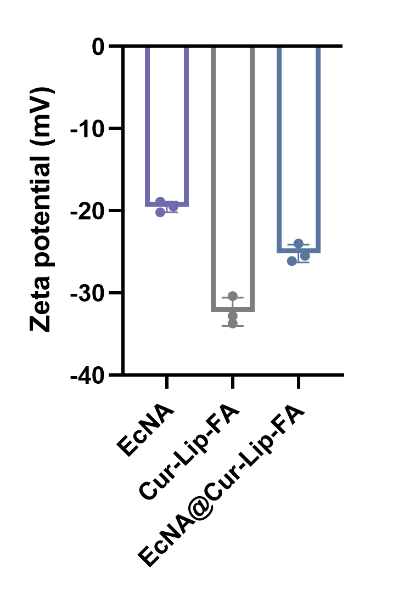


**Figure S6.** Zeta potential of different nanoparticles. Data were shown as the mean ± SD.

**
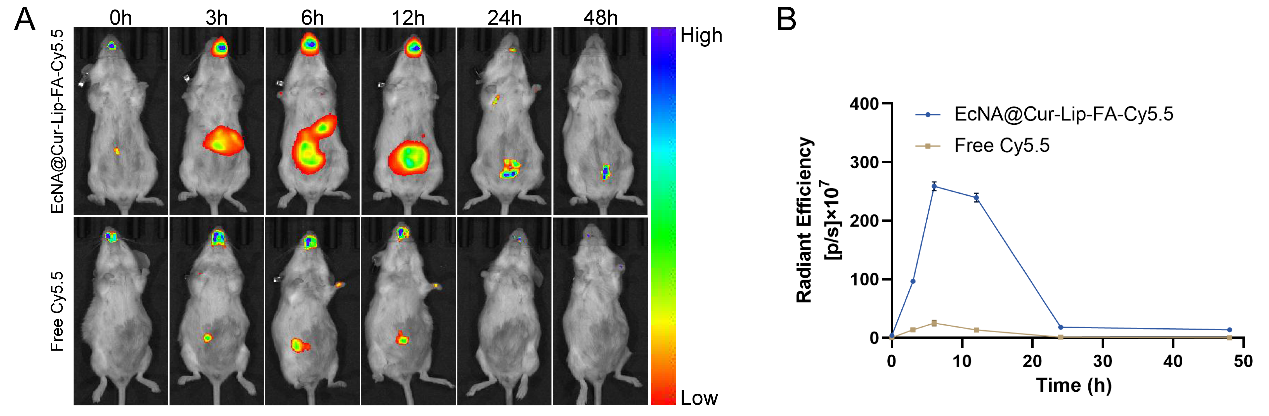
**

**Figure S7.** A) In vivo retention assessment of EcNA@Cur-Lip-FA and B) corresponding quantitative result. Data were shown as the mean ± SD.

**
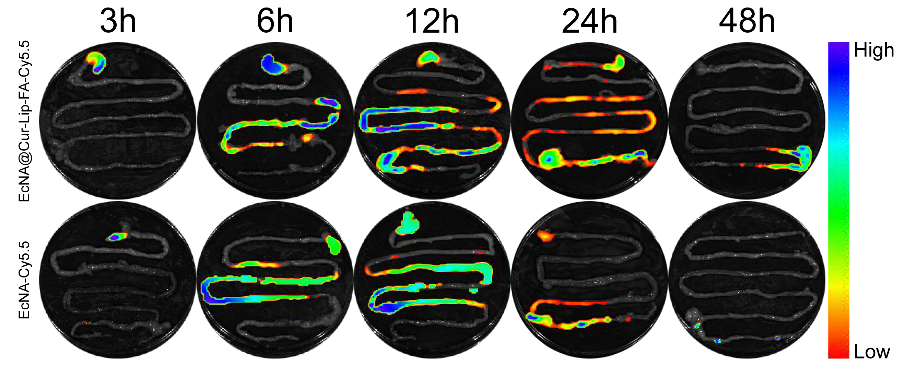
**

**Figure S8.** Intestinal retention assessment of EcNA@Cur-Lip-FA and EcNA.


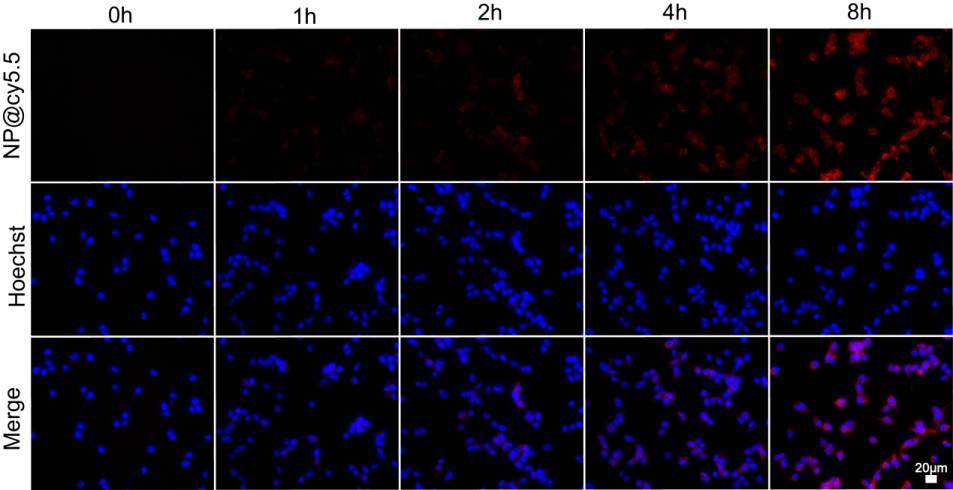


**Figure S9.** Intracellular uptake images of EcNA@Cur-Lip-FA@Cy5.5 at different time points (n = 3, scale bar: 20 μm).


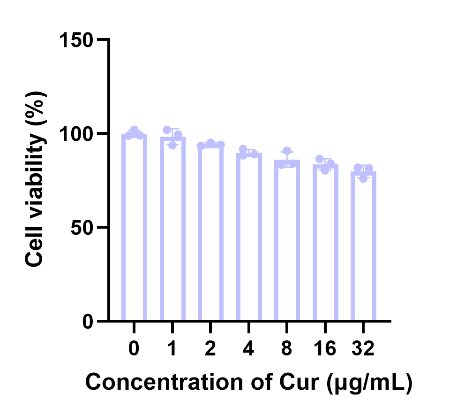


**Figure S10.** Cytotoxicity impact on NCM460 cells (n=3) after administration with different concentrations of EcNA@Cur-Lip-FA + US irradiation. Data were shown as the mean ± SD.


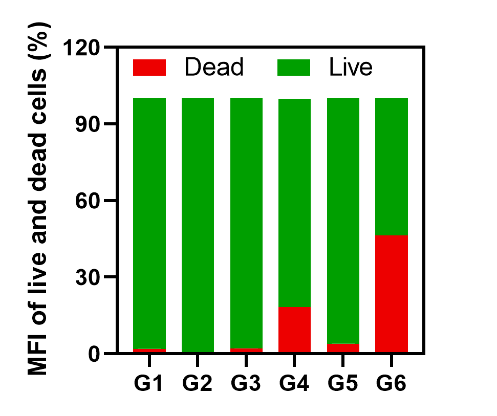


**Figure S11.** MFI ratio of green/red fluorescence images of CT26 cells after different treatments (n=3). Groups are designated to be G1: Control; G2: US; G3: EcNA; G4: Cur-Lip-FA+US; G5: EcNA@Cur-Lip-FA; G6: EcNA@Cur-Lip-FA+US.


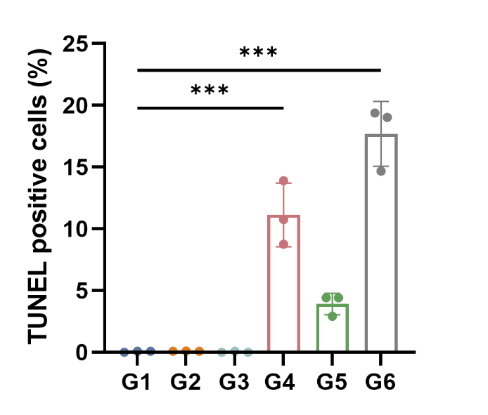


**Figure S12.** The proportion of TUNEL-positive cells (n=3). The statistical method adopted ANOVA analysis. Data were shown as the mean ± SD. *P < 0.05, **P < 0.01 and ***P < 0.001. Groups are designated to be G1: Control; G2: US; G3: EcNA; G4: Cur-Lip-FA+US; G5: EcNA@Cur-Lip-FA; G6: EcNA@Cur-Lip-FA+US.


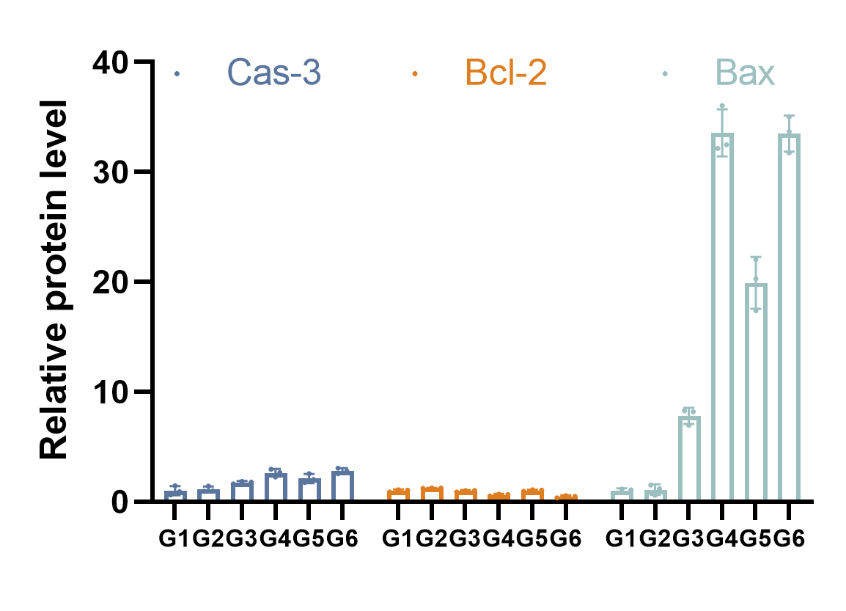


**Figure S13.** WB quantitative analysis of the apoptosis-related proteins. Data were shown as the mean ± SD. Groups are designated to be G1: Control; G2: US; G3: EcNA; G4: Cur-Lip-FA+US; G5: EcNA@Cur-Lip-FA; G6: EcNA@Cur-Lip-FA+US.


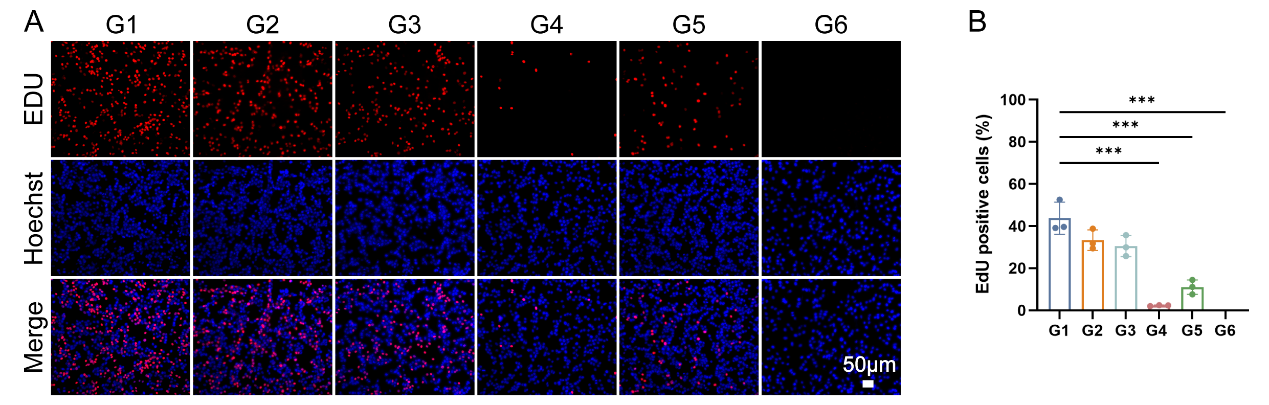


**Figure S14.** EdU fluorescence assay. A) Fluorescence images of EdU positive cells after treatments (n=3, scale bar: 50 μm). B) Quantitative analysis results of EdU fluorescence images (n=3). The statistical method adopted ANOVA analysis. Data were shown as the mean ± SD. *P < 0.05, **P < 0.01 and ***P < 0.001. Groups are designated to be G1: Control; G2: US; G3: EcNA; G4: Cur-Lip-FA+US; G5: EcNA@Cur-Lip-FA; G6: EcNA@Cur-Lip-FA+US.


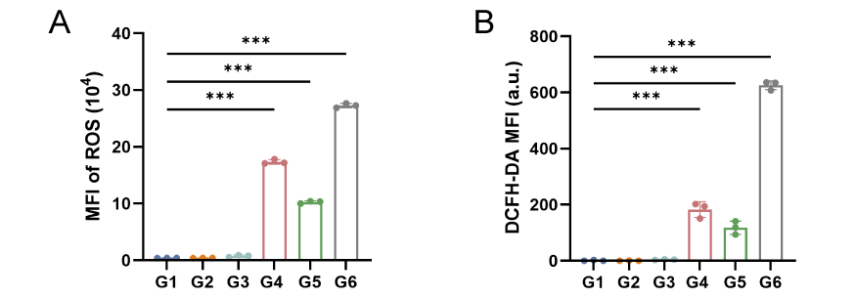


**Figure S15.** Quantitative analysis data of ROS. A) Flow cytometry data of ROS in CT26 cells (n=3). B) Quantitative analysis results of ROS fluorescence images (n=3). The statistical method adopted ANOVA analysis. Data were shown as the mean ± SD. *P < 0.05, **P < 0.01 and ***P < 0.001. Groups are designated to be G1: Control; G2: US; G3: EcNA; G4: Cur-Lip-FA+US; G5: EcNA@Cur-Lip-FA; G6: EcNA@Cur-Lip-FA+US.


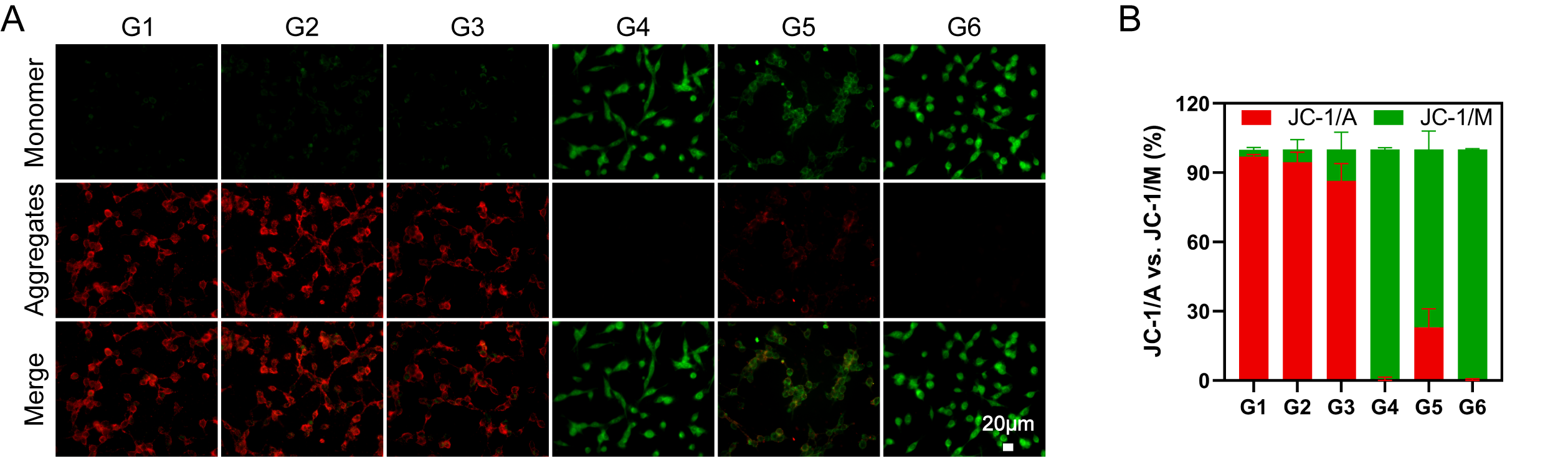


**Figure S16.** JC-1 fluorescence assay. A) Fluorescence images of JC-1A/JC-1/M after different treatments (n=3, scale bar: 20 μm). B) Quantitative analysis results of JC-1A/JC-1/M fluorescence images. Groups are designated to be G1: Control; G2: US; G3: EcNA; G4: Cur-Lip-FA+US; G5: EcNA@Cur-Lip-FA; G6: EcNA@Cur-Lip-FA+US.


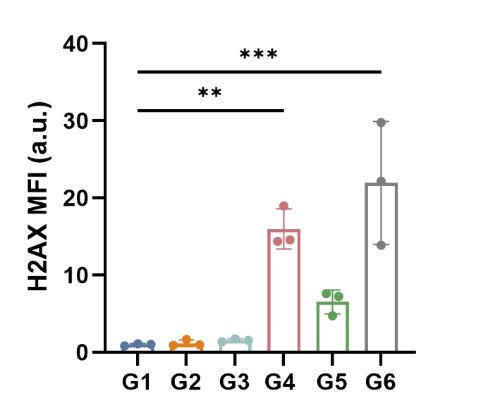


**Figure S17.** Quantitative analysis results of γ-H2AX fluorescence images. (n=3). The statistical method adopted ANOVA analysis. Data were shown as the mean ± SD. *P < 0.05, **P < 0.01 and ***P < 0.001. Groups are designated to be G1: Control; G2: US; G3: EcNA; G4: Cur-Lip-FA+US; G5: EcNA@Cur-Lip-FA; G6: EcNA@Cur-Lip-FA+US.


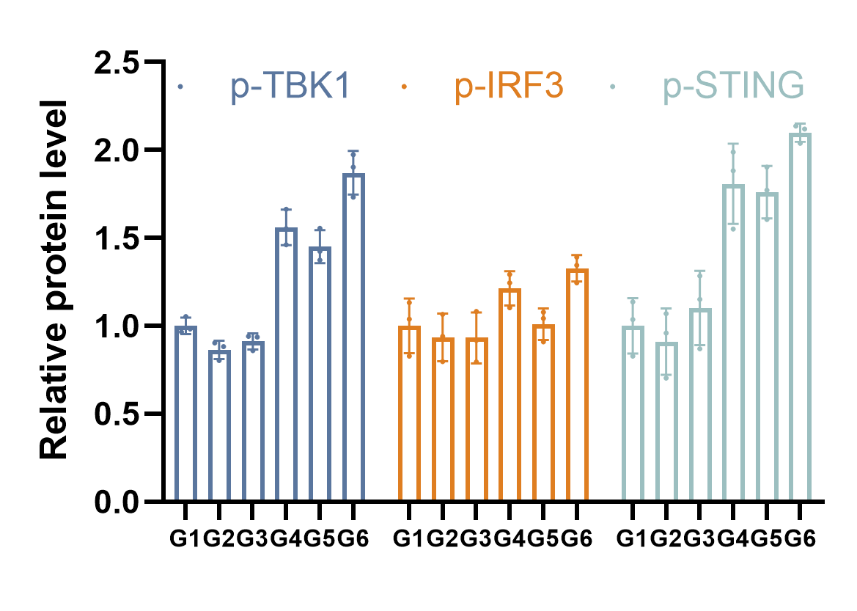


**Figure S18.** WB quantitative analysis of the proteins related to the cGAS-STING pathway. Data were shown as the mean ± SD. Groups are designated to be G1: Control; G2: US; G3: EcNA; G4: Cur-Lip-FA+US; G5: EcNA@Cur-Lip-FA; G6: EcNA@Cur-Lip-FA+US.


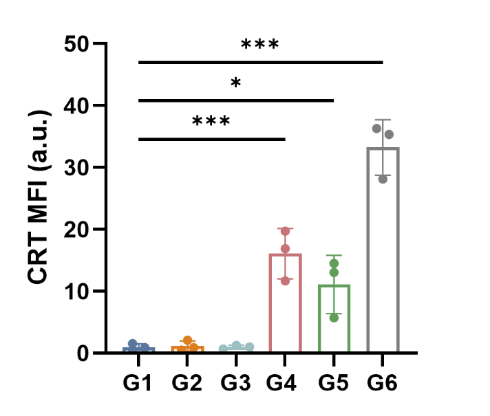


**Figure S19.** Quantitative analysis results of CRT fluorescence images. (n=3). The statistical method adopted ANOVA analysis. Data were shown as the mean ± SD. *P < 0.05, **P < 0.01 and ***P < 0.001. Groups are designated to be G1: Control; G2: US; G3: EcNA; G4: Cur-Lip-FA+US; G5: EcNA@Cur-Lip-FA; G6: EcNA@Cur-Lip-FA+US.


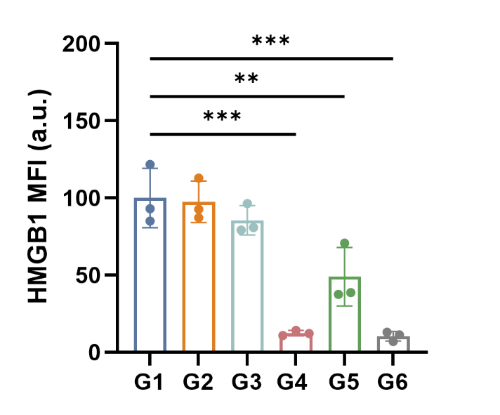


**Figure S20.** Quantitative analysis results of HMGB1 fluorescence images. (n=3). The statistical method adopted ANOVA analysis. Data were shown as the mean ± SD. *P < 0.05, **P < 0.01 and ***P < 0.001. Groups are designated to be G1: Control; G2: US; G3: EcNA; G4: Cur-Lip-FA+US; G5: EcNA@Cur-Lip-FA; G6: EcNA@Cur-Lip-FA+US.


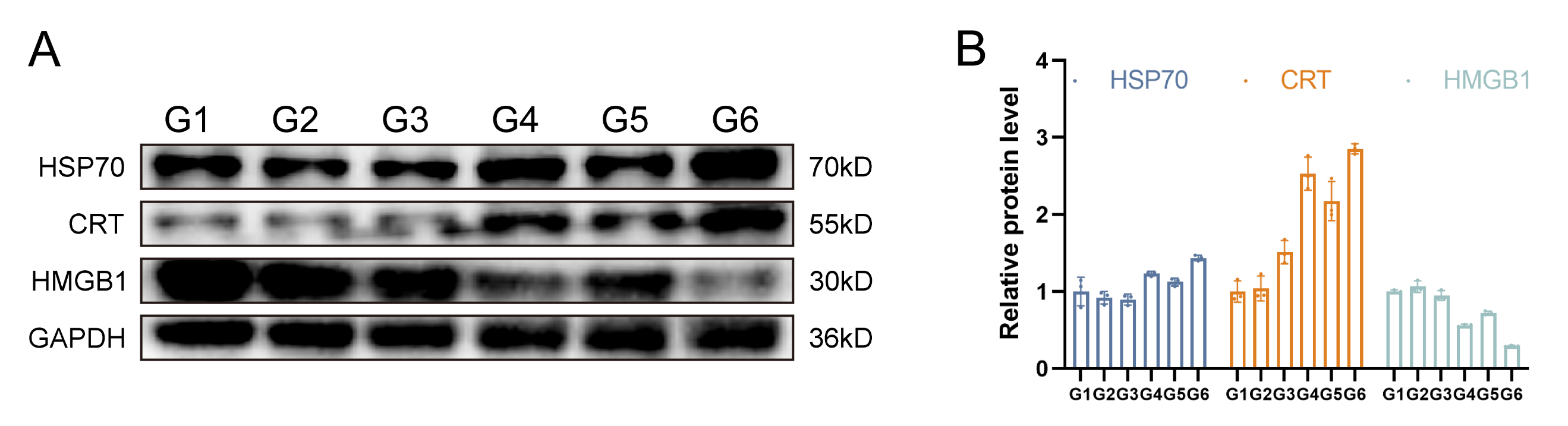


**Figure S21.** A) The expression levels of ICD-related proteins and B) corresponding quantitative result. Data were shown as the mean ± SD. Groups are designated to be G1: Control; G2: US; G3: EcNA; G4: Cur-Lip-FA+US; G5: EcNA@Cur-Lip-FA; G6: EcNA@Cur-Lip-FA+US.


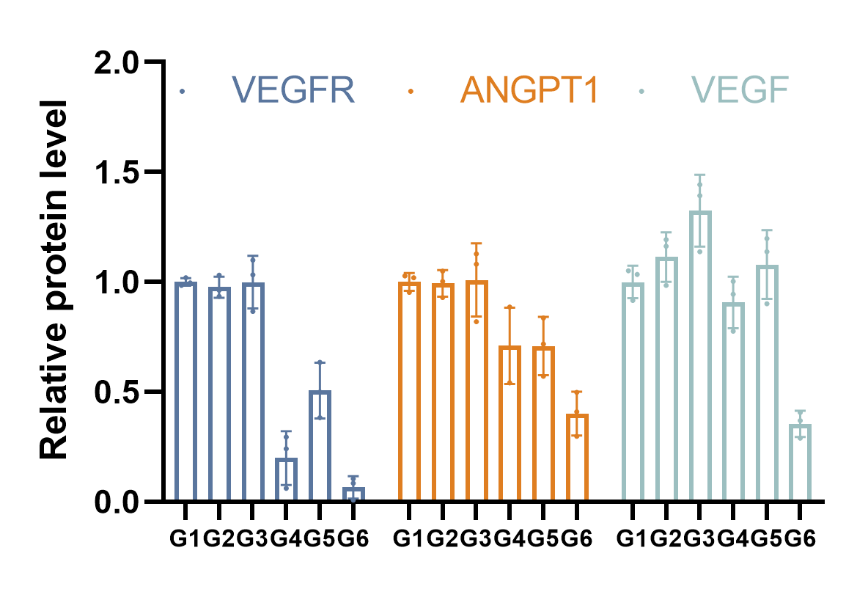


**Figure S22.** WB quantitative analysis of angiogenesis-related proteins. Data were shown as the mean ± SD. Groups are designated to be G1: Control; G2: US; G3: EcNA; G4: Cur-Lip-FA+US; G5: EcNA@Cur-Lip-FA; G6: EcNA@Cur-Lip-FA+US.

**
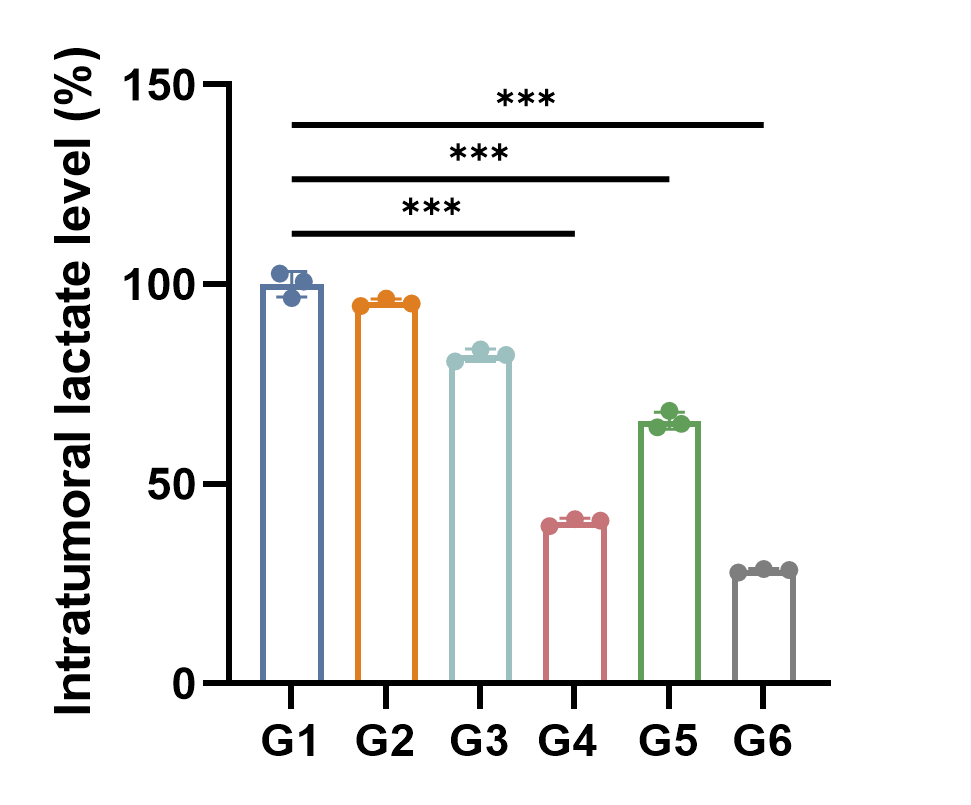
**

**Figure S23.** Intratumoral lactate level. The statistical method adopted ANOVA analysis. Data were shown as the mean ± SD. *P < 0.05, **P < 0.01 and ***P < 0.001. Groups are designated to be G1: Control; G2: US; G3: EcNA; G4: Cur-Lip-FA+US; G5: EcNA@Cur-Lip-FA; G6: EcNA@Cur-Lip-FA+US.


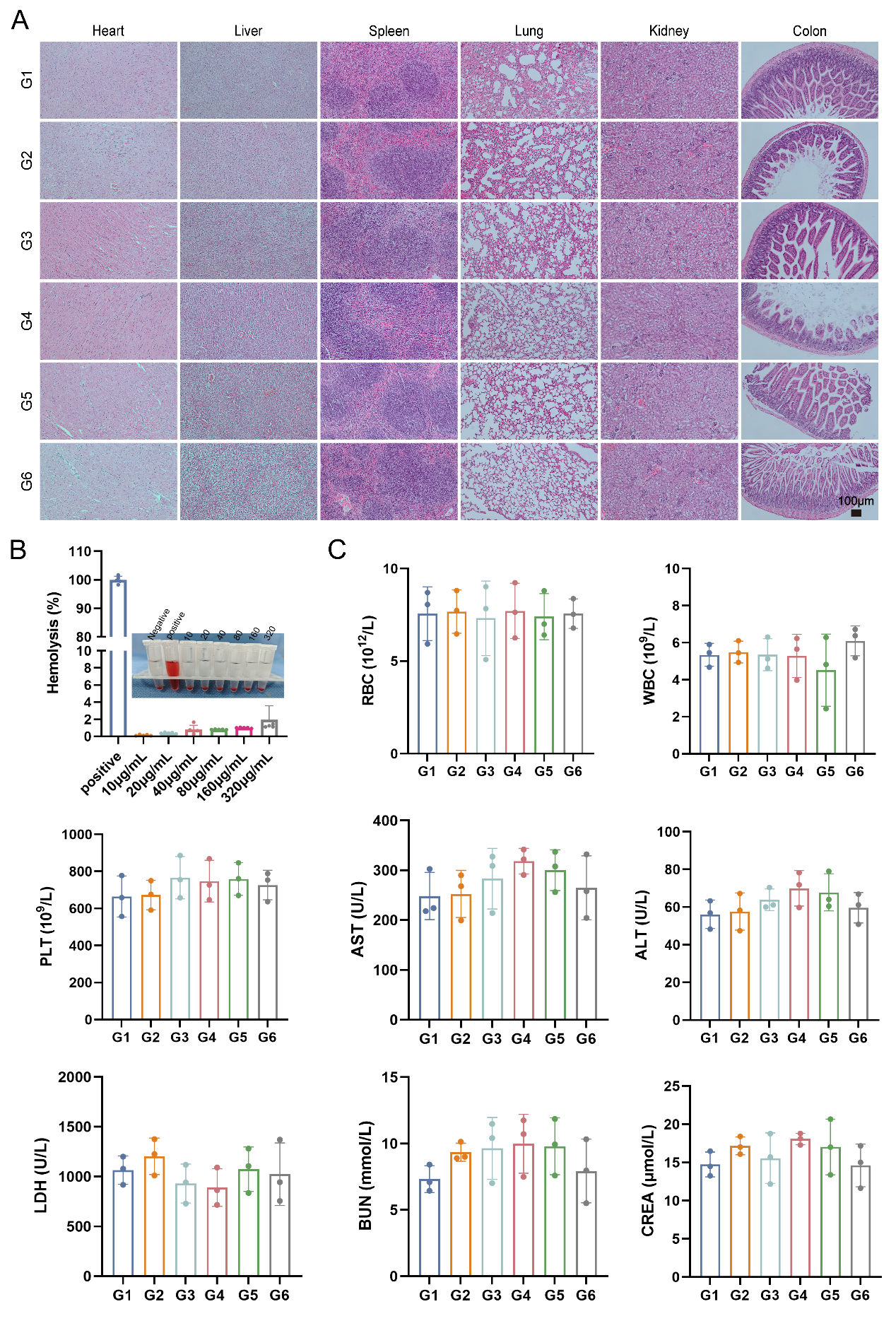


**Figure S24.** Biosafety Assessment of Nanoparticles. A) H&E staining (scales bar =100 μm) of major mouse organs (heart, liver, spleen, lung, kidney, intestine) in different groups. B) Hemolysis analysis. (n=3). C) Blood and serum biochemical data (n=3). Data were shown as the mean ± SD. Groups are designated to be G1: Control; G2: US; G3: EcNA; G4: Cur-Lip-FA+US; G5: EcNA@Cur-Lip-FA; G6: EcNA@Cur-Lip-FA+US.


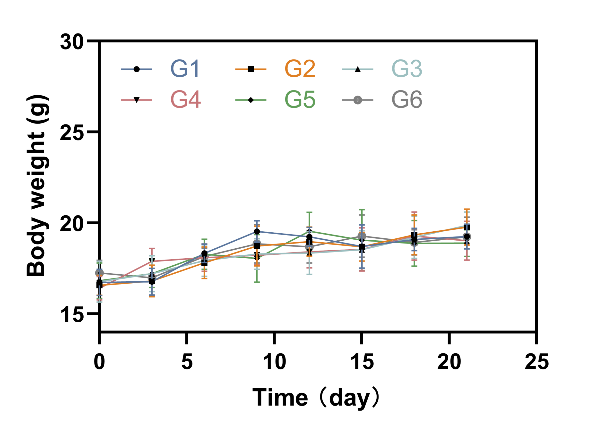


**Figure S25.** Body weights of the CT26 orthotopic cancer mice during the experimental process (n=5). Data were shown as the mean ± SD. Groups are designated to be G1: Control; G2: US; G3: EcNA; G4: Cur-Lip-FA+US; G5: EcNA@Cur-Lip-FA; G6: EcNA@Cur-Lip-FA+US.


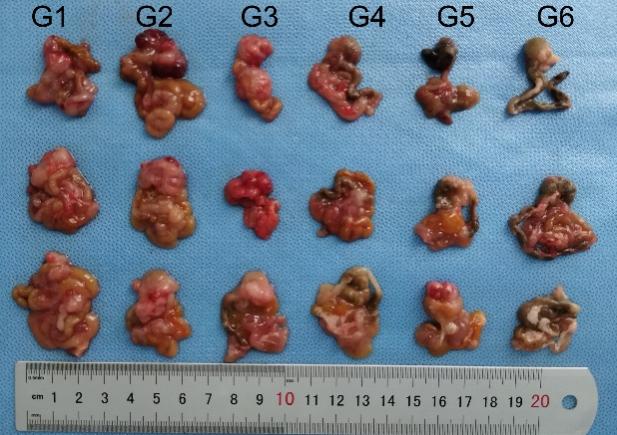


**Figure S26.** Colonic images of mice after different treatments (n=3). Groups are designated to be G1: Control; G2: US; G3: EcNA; G4: Cur-Lip-FA+US; G5: EcNA@Cur-Lip-FA; G6: EcNA@Cur-Lip-FA+US.


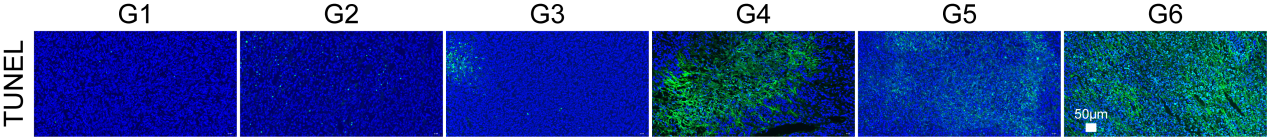


**Figure S27.** TUNEL staining of colonic tissue after different treatments (n=3, scale bar: 50 μm). Groups are designated to be G1: Control; G2: US; G3: EcNA; G4: Cur-Lip-FA+US; G5: EcNA@Cur-Lip-FA; G6: EcNA@Cur-Lip-FA+US.


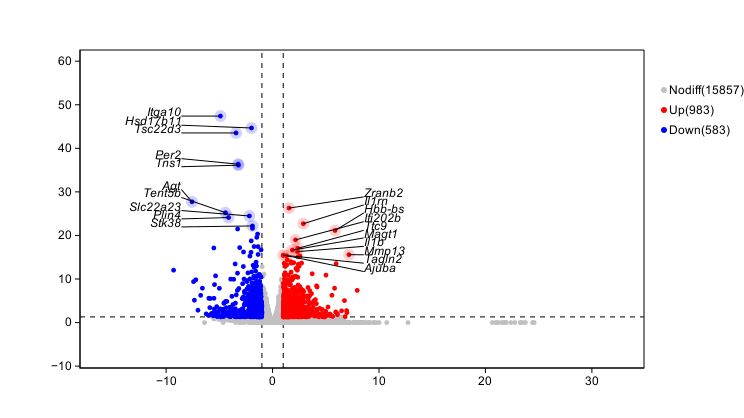


**Figure S28.** The volcano plot showing upregulated and downregulated genes in the Control and EcNA@Cur-Lip-FA+US groups.


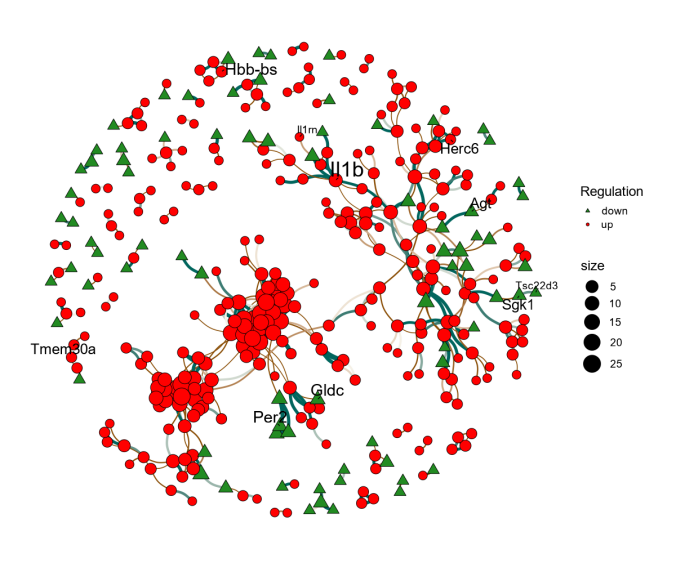


**Figure S29.** Functional interaction network showing close physical interactions among differentially expressed genes.


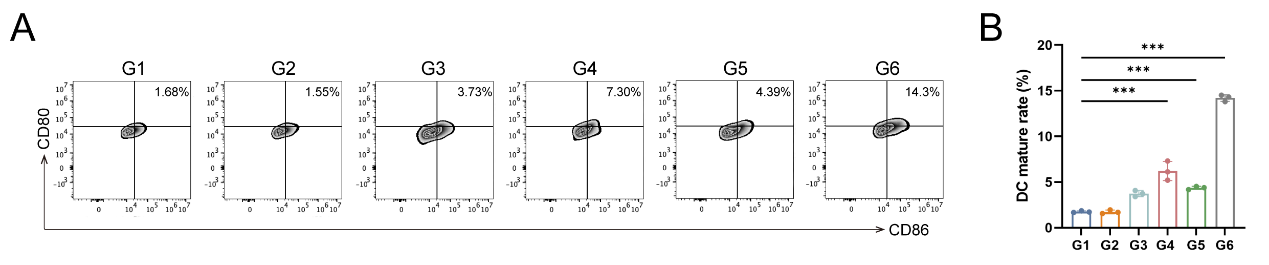


**Figure S30.** Flow cytometry analysis data of DCs at tumor site. A) FCM of DCs at tumor site. B) Quantitative analysis of DCs at tumor site. The statistical method adopted ANOVA analysis. Data were shown as the mean ± SD. *P < 0.05, **P < 0.01 and ***P < 0.001. Groups are designated to be G1: Control; G2: US; G3: EcNA; G4: Cur-Lip-FA+US; G5: EcNA@Cur-Lip-FA; G6: EcNA@Cur-Lip-FA+US.


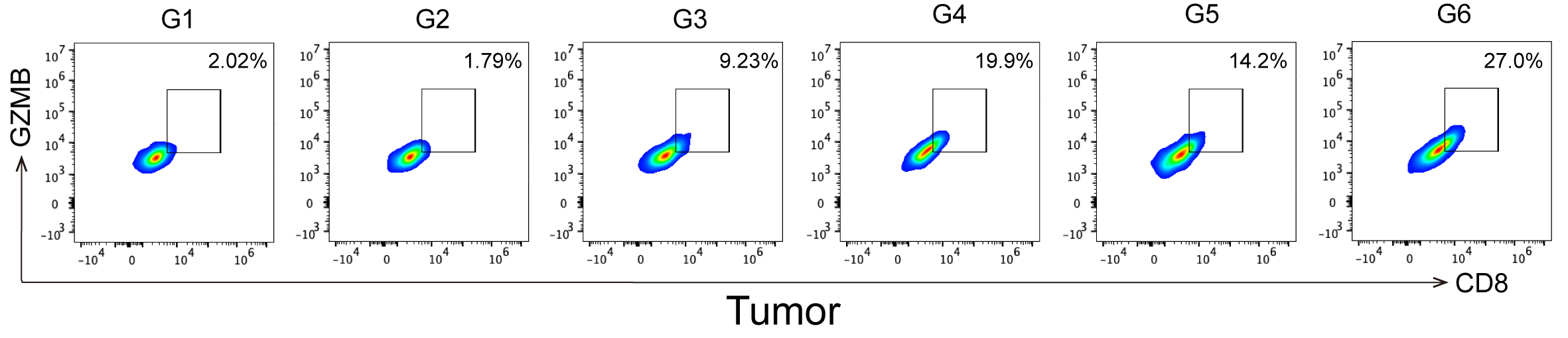


**Figure S31.** FCM of GZMB^+^ in CD8^+^ T cells at tumor site. Groups are designated to be G1: Control; G2: US; G3: EcNA; G4: Cur-Lip-FA+US; G5: EcNA@Cur-Lip-FA; G6: EcNA@Cur-Lip-FA+US.


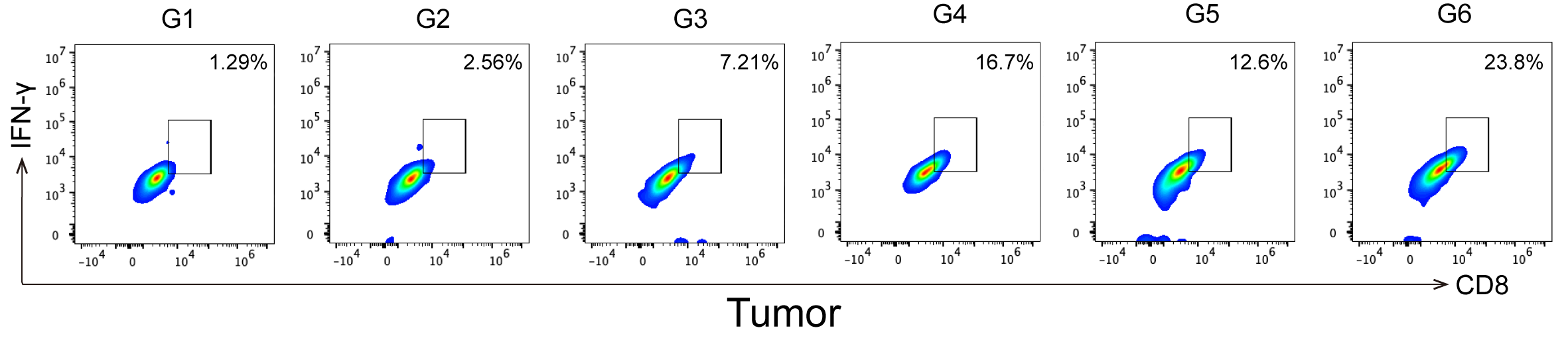
**Figure S32.** FCM of IFN-γ in CD8^+^ T cells at tumor site. Groups are designated to be G1: Control; G2: US; G3: EcNA; G4: Cur-Lip-FA+US; G5: EcNA@Cur-Lip-FA; G6: EcNA@Cur-Lip-FA+US.


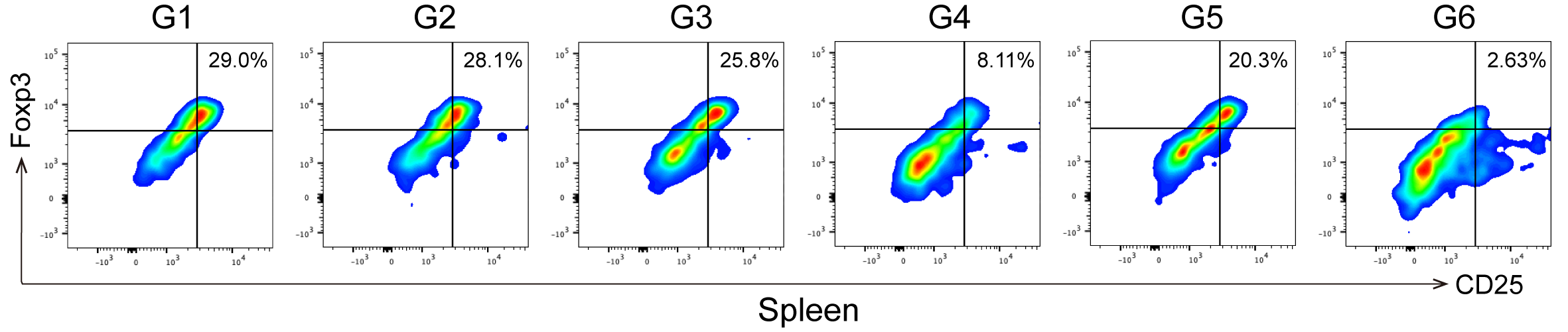


**Figure S33.** FCM of Treg cells in spleen after different treatments. Groups are designated to be G1: Control; G2: US; G3: EcNA; G4: Cur-Lip-FA+US; G5: EcNA@Cur-Lip-FA; G6: EcNA@Cur-Lip-FA+US.


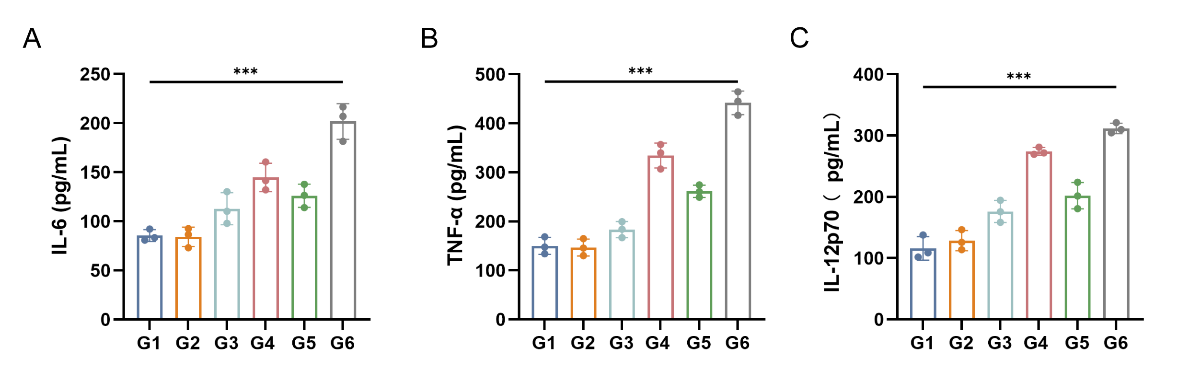


**Figure S34.** (A-C) Serum levels of IL-6, TNF-α and IL-12p70. The statistical method adopted ANOVA analysis. Data were shown as the mean ± SD. *P < 0.05, **P < 0.01 and ***P < 0.001. Groups are designated to be G1: Control; G2: US; G3: EcNA; G4: Cur-Lip-FA+US; G5: EcNA@Cur-Lip-FA; G6: EcNA@Cur-Lip-FA+US.
